# CaMello-XR enables visualization and optogenetic control of G_q/11_ signals and receptor trafficking in GPCR-specific domains

**DOI:** 10.1038/s42003-019-0292-y

**Published:** 2019-02-14

**Authors:** Dennis Eickelbeck, Raziye Karapinar, Alexander Jack, Sandra T. Suess, Ruxandra Barzan, Zohre Azimi, Tatjana Surdin, Michelle Grömmke, Melanie D. Mark, Klaus Gerwert, Dirk Jancke, Petra Wahle, Katharina Spoida, Stefan Herlitze

**Affiliations:** 1 Department of General Zoology and Neurobiology, ND7/31, Ruhr-University Bochum, Universitätsstr. 150, D-44780 Bochum, Germany; 2 Developmental Neurobiology, ND6/72, Ruhr-University Bochum, Universitätsstr. 150, D-44780 Bochum, Germany; 3 Optical Imaging Group, Institut für Neuroinformatik, NB 2/27, Ruhr-University Bochum, Universitätsstr. 150, D-44780 Bochum, Germany; 4 Department of Biophysics, ND04/596, Ruhr-University Bochum, Universitätsstr. 150, D-44780 Bochum, Germany

## Abstract

The signal specificity of G protein-coupled receptors (GPCRs) including serotonin receptors (5-HT-R) depends on the trafficking and localization of the GPCR within its subcellular signaling domain. Visualizing traffic-dependent GPCR signals in neurons is difficult, but important to understand the contribution of GPCRs to synaptic plasticity. We engineered CaMello (Ca^2+^-melanopsin-local-sensor) and CaMello-5HT_2A_ for visualization of traffic-dependent Ca^2+^ signals in 5-HT_2A_-R domains. These constructs consist of the light-activated G_q/11_ coupled melanopsin, mCherry and GCaMP6m for visualization of Ca^2+^ signals and receptor trafficking, and the 5-HT_2A_ C-terminus for targeting into 5-HT_2A_-R domains. We show that the specific localization of the GPCR to its receptor domain drastically alters the dynamics and localization of the intracellular Ca^2+^ signals in different neuronal populations in vitro and in vivo. The CaMello method may be extended to every GPCR coupling to the G_q/11_ pathway to help unravel new receptor-specific functions in respect to synaptic plasticity and GPCR localization.

## Abstract (web summary)

Dennis Eickelbeck et al. engineered light-activated constructs, CaMello and CaMello-5HT_2A_, which are targeted to the 5HT_2A_-R domains and enable visualization of calcium signals and receptor trafficking in response to activation. The reported CaMello tool could be applied to other GPCRs coupled to the G_q/11_ signaling pathways which may shed light on mechanisms of GPCR localization and plasticity.

## Introduction

Changes in the intracellular Ca^2+^ concentration in neurons regulate various cellular processes including synaptic transmitter release, gene transcription, and various forms of synaptic plasticity^1^. These Ca^2+^ signals are spatio-temporally controlled in their amplitude and can occur as fast Ca^2+^ spikes or Ca^2+^ oscillations^2^. Many Ca^2+^ signaling molecules are assembled into macromolecular complexes in specific subcellular microdomains, which function autonomously within highly specialized environments^1^. An example for such a functional subcellular microdomain is the assembly of voltage gated Ca^2+^ channels with the transmitter release machinery at the presynaptic terminal^3^.

Transmitter-mediated increases in intracellular Ca^2+^ levels not only involve the fast gating of plasma membrane ion channels but also GPCRs, coupling to the G_q/11_ pathway. Ion channels and GPCRs are activated by various transmitters such as glutamate, histamine, oxytocin or serotonin, where the Ca^2+^ signaling components are often assembled into spatially separated signaling complexes.

For example, metabotropic mGluRs assemble into a macromolecular complex with IP_3_ receptors via the scaffolding protein Homer and co-purify with protein phosphatases and protein kinase A (PKA)^1,4^. Ca^2+^ release from internal stores in neurons is regulated via the activation of phospholipase C (PLC), hydrolysis of phosphatidylinositol 4,5-bisphosphate (PIP_2_) into diacylglycerol (DAG) and inositol 1,4,5-trisphosphate (IP_3_) and activation of IP_3_ receptors located at the endoplasmic reticulum (ER), which leads to the release of Ca^2+^ from the ER^5^. The opening of small groups of IP_3_ receptors induces a puff of Ca^2+^. Summation of several of these puffs can elicit an intracellular Ca^2+^ wave^3^. Depending on which isoforms of the different signaling components are activated different forms of Ca^2+^ signals are induced in neurons. For instance, activation of mGluR1 in neurons produces a single Ca^2+^ transient, whereas mGluR5 generates an oscillatory Ca^2+^ wave^1,6^.

5-HTRs coupling to the G_q/11_ pathway such as 5-HT_2A/C_-Rs are abundantly expressed in the brain and are molecular targets for atypical antipsychotic drugs and most hallucinogens^7^. 5-HT_2A_-Rs are expressed on apical dendrites of cortical pyramidal neurons and cerebellar Purkinje cells. 5-HT_2A_-Rs colocalize with PSD95 and MUPP1 (multi-PDZ domain protein 1) in apical dendrites, dendritic shafts and spines^8^. The targeting and subcellular localization of 5-HT_2A_-Rs involve a PDZ binding domain in the C-terminus (CT) of the 5-HT_2A_-R^9,10^. Activation of 5-HT_2A_ has been associated with changes in spine and dendritic morphology^8^, changes in BDNF levels in the hippocampus and neocortex^11^ and normally results in an increased neuronal activity^12,13^. It has been suggested that increased activity of 5-HT_2A_-Rs might be responsible for some of the psychotic symptoms in schizophrenia^14^ and that atypical antipsychotic agents may antagonize the hyperactivity and membrane targeting of 5-HT_2A_-Rs^15^. In addition, depending on the agonist and cell-type 5-HT_2A_-Rs not only stimulate the Gq-PLC pathway, but also other pathways such as the G_12/13_-PLA_2_ and G_i/o_-Src pathway^16–18^. These observations suggest that alterations in 5-HT_2A_-R trafficking and G protein signaling contribute to the development and manifestation of neuropsychiatric disorders.

Thus, decoding Ca^2+^ signals in GPCR-specific microdomains is important for understanding the functions of GPCRs in their native environment. It is also important to understand how these signals are shaped by GPCR trafficking and internalization, how they contribute to neuronal excitation and plasticity and how these signals are altered under pathological conditions. We therefore engineered CaMello-XR and mloCal-XR to optogenetically control and monitor the intracellular Ca^2+^ changes directly in the GPCR microdomain and relate the Ca^2+^ signal to the trafficking of the GPCR. While mloCal-XRs consist of a membrane-localized calcium sensing domain combined with a second spectrally shifted fluorescent tag and a receptor trafficking signal, allowing for passive visualization of receptor trafficking and intracellular Ca^2+^ signals in receptor-specific domains, CaMello-XRs are engineered in a comparable manner, but are built around a light-activated GPCR as a backbone and thus allow for additional optogenetic control of G_q/11_ signals. While this technique can most likely be applied to every GPCR coupling to the G_q/11_ pathway, we established the method using the 5-HT_2A_-R because of its importance for neuropsychiatric disorders and the lack of understanding of how this GPCR modulates neuronal activity and plasticity.

## Results

### Visualization of GPCR trafficking and intracellular Ca^2+^ signals

**Fig. 1:**
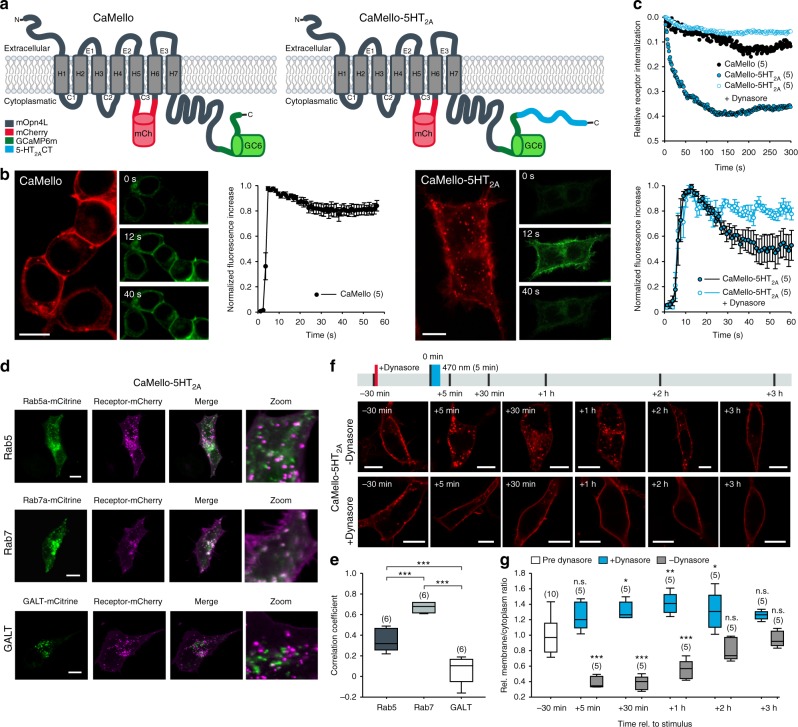
Optogenetic control and visualization of Ca^2+^ signals with CaMello-XRs. **a** CaMello and CaMello-5HT_2A_ design. Both chimeric constructs consist of mouse melanopsin (mOpn4L), mCherry inserted into C3 and GCaMP6m added to the CT. CaMello-5HT_2A_ additionally has the 5-HT_2A_ receptor CT appended (C: intracellular loop; CT: C-terminus; H: transmembrane helix; E: extracellular loop). **b** Time course of light-induced Ca^2+^ responses in HEK tsA201 cells. Transfected cells were visualized (mCherry, 561 nm) and Ca^2+^ signals were measured (GCaMP6m, 476 + 495 nm) (images). Normalized Ca^2+^ responses during 60 s of illumination (graphs), for CaMello-5HT_2A_ with/without addition of dynamin inhibitor Dynasore (50 µM) (mean ± s.e.m.; *n* = 5 dishes). Scale bar, 10 µm. **c** Normalized activation-dependent receptor internalization monitored via differences in membrane-localized mCherry fluorescence reduction between stimulated (476 nm + 561 nm) and unstimulated (561 nm) trials, for CaMello-5HT2A with/without addition of Dynasore (50 µM) (mean; *n* = 5 dishes). **d** Colocalization of CaMello-5HT_2A_ with Rab5a (-mCitrine, early endosome), Rab7a (early to late endosome) or GALT (beta-1,4-galactosyltransferase 1, trans-Golgi network) 5 min post stimulation with 476 nm light (5 min). Scale bar, 10 µm. **e** Averages of the calculated Pearson’s correlation coefficient for the colocalization as shown in d (box plot; one-way analysis of variance (ANOVA) and Holm-Sidak multiple comparison method; *n* = 6 individual cells for each colocalization pairing; ***p < 0.001). **f** Time course (confocal z-sections) of activation-dependent receptor internalization/recycling for CaMello-5HT_2A_. Cells were stimulated with blue light (476 nm, 5 min) at 0 min and receptor internalization/recycling was monitored over time. At −30 min Dynasore (50 µM) was added to the culture medium (control group). Scale bar, 10 µm. **g** Relative membrane to cytoplasm ratio as seen in (**f**) was calculated at indicated time points via quantitative mCherry fluorescence intensity analysis (box plot; one-way repeated measures analysis of variance (RM ANOVA) versus control and Holm-Sidak multiple comparison method; *n* = (*x*), # of individual cells per group; n.s. = not significant,*p < 0.05, **p < 0.01, ***p < 0.001; *p* left to right: 0.064, < 0.001, 0.037, < 0.001, 0.003, < 0.001, 0.036, 0.081, 0.101, 0.393)

In order to engineer a light-activated GPCR for the visualization of receptor trafficking and intracellular Ca^2+^ signals we introduced the red fluorescent protein mCherry (mCh) into the III intracellular loop of the light-activated GPCR melanopsin from mouse (mOpn4L or moMo). In addition, we fused GCaMP6m^19^ to the CT of moMo. We call this construct CaMello (Ca^2+^-melanopsin-local-sensor, Fig. 1a). Expression of CaMello in HEK tsA201 cells and illumination of the cell with red light reveals the localization of the GPCR at the plasma membrane by the intracellular mCh tag. Activation of CaMello by blue light leads to an immediate increase in intracellular Ca^2+^ signal detected by the C-terminally localized GCaMP6m. The Ca^2+^ signal is sustained during receptor activation^20^ (Fig. 1b, Supplementary Movie 1) and correlates with the stable fluorescent mCh signal at the membrane (Fig. 1c) suggesting that CaMello is not internalized during G_q/11_ activation.

In former studies we showed that the CT of GPCRs such as the 5-HT_1A_-R, 5-HT_1B_-R, and 5-HT_2C_-R is sufficient for localization of the GPCR into the receptor-specific domain in neurons^21–24^. Thus, in order to analyze GPCR-specific G_q/11_ signals in the brain we tagged CaMello with the CT of the 5-HT_2A_-R at the C-terminal end and analyzed if the 5-HT_2A_-CT could change GPCR trafficking and G_q/11_ induced Ca^2+^ signals. This construct is called CaMello-5HT_2A_. The 5-HT_2A_-R is a G_q/11_ coupled GPCR, which is expressed throughout the brain, involved in various psychiatric disorders such as schizophrenia, anxiety, and depression, activated by hallucinogens and a drug target for various anxiolytics, antidepressants, and antipsychotics^25^. As detected by the mCh tag our engineered GPCR is localized to the membrane and to intracellular vesicle like structures. Blue light-mediated activation of CaMello-5HT_2A_ leads to a fast rise in intracellular Ca^2+^ levels, which declines to a steady state level at about 50% of the maximal response amplitude (Fig. 1b, Supplementary Movie 2). The decline in response amplitude during receptor activation correlates with a decrease in the fluorescent mCh signal at the plasma membrane (Fig. 1c). The decline in mCh and Ca^2+^ signals during light activation of CaMello-5HT_2A_ are blocked by Dynasore (Fig. 1b, c), an inhibitor of dynamin-dependent endocytosis^26,27^. Five-minute light stimulation of CaMello-5HT_2A_ leads to an increase of cytoplasmic (vesicle) localization relative to transmembrane localization, which is recovered after 2 h (Fig. 1d). The increase in cytoplasmic vesicle localization is blocked by Dynasore (Fig. 1d). In addition, the 5-HT_2A_-R and CaMello-5HT_2A_ colocalize with early (Rab5) and late (Rab7) endosome markers, but not with the Golgi marker GALT. The relative decline in GPCR localization at the plasma membrane after GPCR activation is comparable to the mCh tagged 5-HT_2A_-R, which also colocalizes with the Rab5 and Rab7, but not with GALT (Supplementary Figure 1). The colocalization between 5-HT_2A_-R and the endosome markers may depend on the ligand/ligand concentration as has been suggested in various studies (see for example, refs. ^28–30^). Indeed, we found that 5-HT_2A_-R and CaMello-5HT_2A_ stimulated by 1 µM 5-HT or low intensity light are colocalized to a higher degree with the early endosome marker Rab5, while 5-HT_2A_-R and CaMello-5HT_2A_ stimulated by 20 µM TCB-2 or high intensity light are colocalized to a higher degree with the late endosome marker Rab7 (Fig. 1, Supplementary Figures 1 and 2). The results suggest that the decline in Ca^2+^ response is mediated by receptor internalization and depends on dynamin, as has been described for the 5-HT_2A_-R^31^.

**Fig. 2:**
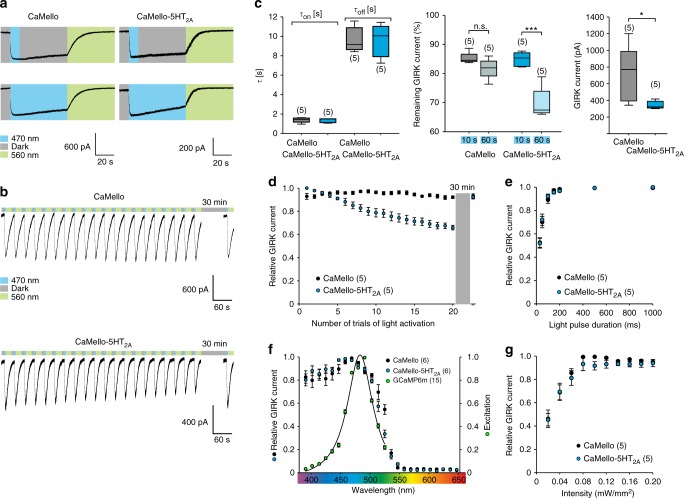
Electrophysiological characterization of CaMello and CaMello-5HT_2A_. **a** Whole-cell patch clamp recordings of light-induced GIRK currents in HEK GIRK 1/2 cells activated and subsequently deactivated via CaMello or CaMello-5HT_2A_ using a 10 s light pulse with a 50 s dark phase (top) or a 60 s light pulse (bottom) of 470 nm for activation followed by a 50 s light pulse of 560 nm for deactivation. **b** Repetitive activation/deactivation of GIRK currents via CaMello or CaMello-5HT_2A_ followed by a 30 min dark phase and an additional activation/deactivation. **c** Time constants of GIRK current activation/deactivation by CaMello/CaMello-5HT_2A_ using 10/50 s light pulses of 470/560 nm (left). Remaining GIRK current after 60 s of recording following 10 or 60 s of 470 nm light stimulation for CaMello/CaMello-5HT_2A_ (middle). Maximal induced GIRK current amplitude for CaMello/CaMello-5HT_2A_ using a 10 s light pulse of 470 nm (right) (box plot; one-way analysis of variance (ANOVA) and Holm-Sidak multiple comparison method or Mann–Whitney rank sum test; *n* = 5 individual cells recorded; n.s. = not significant, *p < 0.05, ***p < 0.001; *p* left to right: 0.889, < 0.001, 0.032). **d** Relative GIRK current response during repetitive light stimulation as shown in (**b**). **e** Light-pulse duration dependence of relative GIRK current activation by CaMello/CaMello-5HT_2A_ using a 470 nm light pulse of the indicated duration for activation followed by a 560 nm light pulse for deactivation. **f** Wavelength dependence of relative GIRK current activation via CaMello/CaMello-5HT_2A_ using a 1 s light pulse of the indicated wavelength for activation followed by a 560 nm light pulse for deactivation. The superimposed (CaMello-)GCaMP6m excitation spectrum was measured via 1 s 470 nm activation of CaMello followed by fluorescence emission recording (530–550 nm) for each indicated excitation wavelength (390–520 nm). **g** Intensity dependence of relative GIRK current activation via CaMello/CaMello-5HT_2A_ using a 1 s 470 nm light pulse of the indicated intensity for activation followed by a 560 nm light pulse for deactivation after each activation. Plotted data (**d**–**g**) presented as mean (± s.e.m); *n* = number of individual cells recorded

We next analyzed the light-dependent activation and deactivation properties of CaMello and CaMello-5HT_2A_ and how wavelength-dependent light activation of the GPCRs overlaps with the visualization of the GCaMP Ca^2+^ signal (Fig. 2). The time constants of light activation and deactivation (Fig. 2c), the light-pulse dependence (Fig. 2e), wavelength dependence (Fig. 2f), and light intensity of GIRK channel activation does not differ between CaMello and CaMello-5HT_2A_, and are comparable to the biophysical properties of moMo (see ref. ^20^). In contrast, repetitive and long-term stimulation of CaMello-5HT_2A_ leads to a 30% decrease in response amplitude, which is not observed for CaMello (Fig. 2a–d). The decline in response amplitude for CaMello-5HT_2A_ is fully recovered after 30 min (Fig. 2b, d). The light-dependent activation of CaMello and CaMello-5HT_2A_ overlap with the excitation peak of GCamP6m (Fig. 2f) allowing for visualization of Ca^2+^ signals during GPCR activation.

In summary, tagging CaMello with the CT of the 5-HT_2A_-R alters the receptor localization and trafficking of the GPCR. This consequentially changes the sustained intracellular Ca^2+^ dynamics that are typically elicited via activation of moMo (or CaMello) by adding a new regulatory mechanism, leading to an altered shape of these dynamics depending on cell-type, domain, and cellular state.

### Receptor-specific Ca^2+^ dynamics in cortical neurons

GPCR/G_q11_-mediated Ca^2+^ signals in neurons have been shown to be involved in modulation of neuronal activity, synaptic plasticity and in processes such as learning and memory^1^. GPCRs can be localized in specific subcellular microdomains and compartments. Therefore, GPCR induced intracellular Ca^2+^ signals may differ in their localization, amplitude, and spatio-temporal distribution in neurons^32^. Hence, we investigated and compared GPCR/G_q/11_ activation and Ca^2+^ dynamics in cortical neurons from organotypic slice cultures (OTCs) using non-targeted and 5-HT_2A_-R domain targeted CaMellos.

**Fig. 3:**
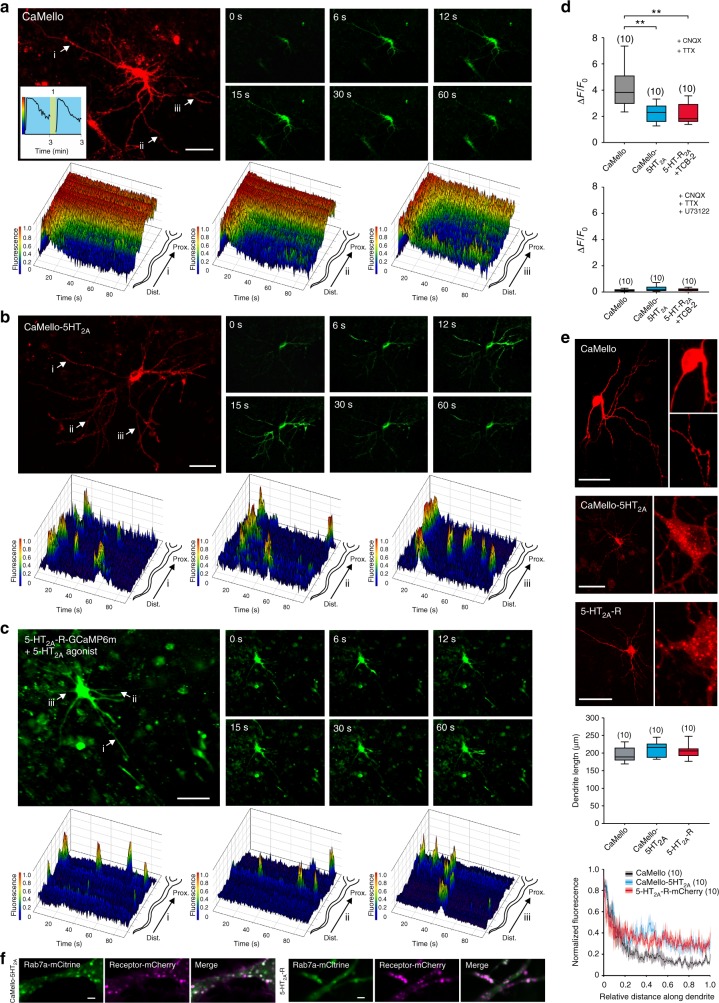
Optogenetic control and visualization of local Ca^2+^ signals in rat visual cortex organotypic cultures (OTCs). **a**–**c** Time course of light-induced (**a**, **b**) and agonist-induced (**c**) local Ca^2+^ responses in rat visual cortex OTCs. Transfected cells were visualized using the mCherry reporter (CaMello + CaMello-5HT_2A_) or GCaMP6m (5-HT_2A_-R) and Ca^2+^ signals were light-induced (CaMello + CaMello-5HT_2A_, 476 + 495 nm) or agonist-induced (5-HT_2A_ receptor, TCB-2 20 µM) and measured via GCaMP6m monitoring (images). 3D mesh plots of individual neurites (i, ii, iii) showing normalized local light-induced (CaMello + CaMello-5HT_2A_) or agonist-induced (5-HT_2A_-R) Ca^2+^ responses during 90 s of illumination from distal to proximal (plots). Repetitive activation and deactivation (561 nm) of CaMello induced Ca^2+^ responses (inset). Scale bar, 50 µm. **d** Comparison of maximal change in induced fluorescence intensity (∆*F*/*F*_0_). Maximal change in ∆*F*/*F*_0_ in the presence of CNQX (1 µM) and TTX (1 µM) (box plots; one-way analysis of variance (ANOVA) and Holm-Sidak multiple comparison method; *n* = 10 individual cells, pooled from five animals per group; **p < 0.01; *p* left to right: 0.001, 0.001) (top). Maximal change in ∆*F*/*F*_0_ in the presence of CNQX (1 µM), TTX (1 µM) and PLC antagonist U73122 (10 µM) (box plots; *n* = 10 individual cells, pooled from five animals per group) (bottom). **e** Differential expression pattern of CaMello, CaMello-5HT_2A_, and the 5-HT_2A_-R mCherry constructs in rat cortical neurons (OTCs). Receptor expression pattern was visualized using the mCherry reporter (561 nm) (images). Normalized fluorescence of the longest dendrite (length = box plot) was plotted against the length of each dendrite (plot) (box plots; *n* = 10 individual cells, pooled from five animals per group). Scale bar, 50 µm. **f** Colocalization of CaMello-5HT_2A_ and the 5-HT_2A_-R mCherry construct with Rab7a in OTCs of the rat visual cortex as seen in Fig. 1 and Supplementary Figure 1 for HEK cells. Scale bar, 2 µm

Expression of CaMello in cortical neurons leads to a strong localization throughout the soma and weak distribution in the distal dendrites (Fig. 3a, e and Supplementary Figure 3). Light activation leads to a sustained increase in intracellular Ca^2+^ levels at the soma and proximal dendrites, and a decline of the Ca^2+^ signal in distal dendrites (Fig. 3b, Supplementary Movies 3, 4). Ca^2+^ responses can be terminated by yellow light and can be activated again after 1 min by blue light (Fig. 3a inset). The Ca^2+^ response measured over the whole neuron declines in amplitude during a 3 min light activation to ~60% (Fig. 3a inset). Throughout the OTC experiments all neurons within the visual field of the microscope were illuminated by light to activate light-activated GPCRs and monitor GCaMP signals.

In contrast, CaMello-5HT_2A_ is localized close to the plasma membrane and is found in intracellular compartments (Fig. 3e and Supplementary Figure 3). The intracellular clusters colocalize with the endosome marker Rab7 (Fig. 3f). CaMello-5HT_2A_ distributes in a comparable manner to proximal and distal dendrites and is also found in thin, spineless neurites with 90° branches, which are most likely axons according to morphological criteria (Supplementary Figure 4)^33^. Endogenous 5-HT_2A_-Rs and mCh or GCaMP6m tagged 5-HT_2A_-Rs reveal a similar distribution as CaMello-5HT_2A_ and are found at the plasma membrane, intracellular compartments, proximal and distal dendrites, the axon and colocalize with GFP tagged postsynaptic density protein 95 (PSD-95) (Fig. 3c, Supplementary Figure 3 and 4). Light activation of CaMello-5HT_2A_ leads to transient Ca^2+^ responses, which occur at different receptor domains randomly distributed throughout the distal and proximal dendrites (Fig. 3b, Supplementary Movie 3, 5). These transient, randomly distributed Ca^2+^ responses are abolished and converted to a global activation of intracellular Ca^2+^ responses after the application of Dynasore (Supplementary Figure 5).

Similar Ca^2+^ responses were observed for mCh and GCaMP6m tagged 5-HT_2A_ receptors (note, 5-HT_2A_-GCaMP6m does not contain mCh in the intracellular loop III) expressed in OTCs (Fig. 3c, e, Supplementary Movie 3, 6). Ca^2+^ responses in average are larger for CaMello in comparison to CaMello-5HT_2A_ as expected from the 5-HT_2A_-R-mediated internalization (Supplementary Figure 1) and are blocked by the PLC antagonist U73122, suggesting that Ca^2+^ responses are activated via the G_q/11_ pathway.

**Fig. 4:**
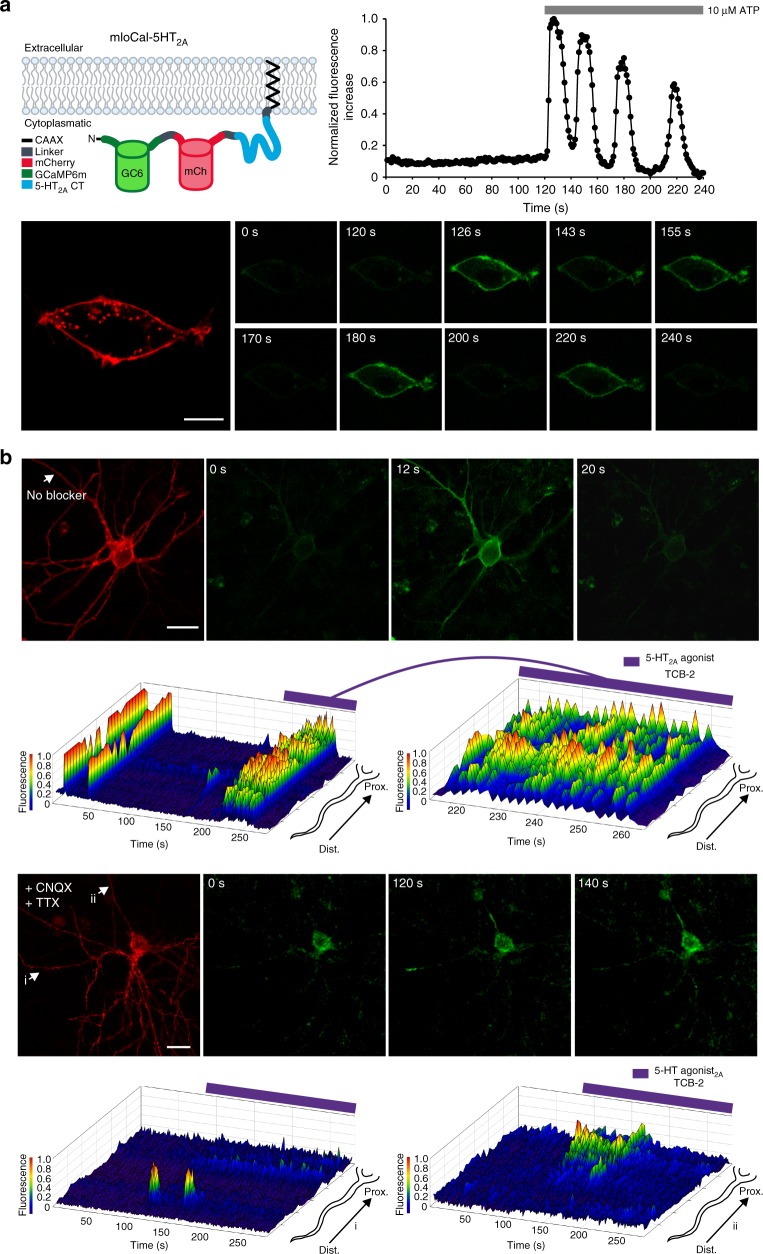
Visualization of local Ca^2+^ signals in HEK cells and rat visual cortex organotypic cultures (OTCs) induced by endogenous GPCRs using mloCal-5HT_2A_. **a** mloCal-5HT_2A_ design. The construct consists of an N-terminal GCaMP6m linked to mCherry, the 5-HT_2A_ receptor C-terminus (CT) and a C-terminal CAAX membrane anchor (human KRAS isoform B C-terminal domain) (schematic). Time course of ATP-induced (10 µM) membrane-localized Ca^2+^ responses in HEK tsA201 cells transfected with mLocal-5HT_2A_ (graph). Cells were visualized using the mCherry reporter (561 nm) and ATP-induced Ca^2+^ signals were measured via GCaMP6m monitoring (476 + 495 nm) (images). Scale bar, 10 µm. **b** Time course of neuronal network driven and 5-HT_2A_-R agonist modulated (TCB-2 20 µM) local Ca^2+^ responses in rat visual cortex OTCs without network blockers. Transfected cells were visualized using the mCherry reporter (561 nm) and Ca^2+^ signals were measured via GCaMP6m monitoring (476 + 495 nm) (top images). 3D mesh plot of an individual neurite (arrow) showing normalized local Ca^2+^ responses during 280 s of illumination with additional 5-HT_2A_ agonist stimulation after 180 s from distal to proximal (top plots). Time course of 5-HT_2A_ agonist modulated (TCB-2 20 µM) local Ca^2+^ responses in rat visual cortex OTCs in the presence of CNQX (1 µM) and TTX (1 µM). Transfected cells were visualized using the mCherry reporter (561 nm) and Ca^2+^ signals were measured via GCaMP6m monitoring (476 + 495 nm) (bottom images). 3D mesh plots of individual neurites (i, ii) showing normalized local Ca^2+^ responses during 280 s of illumination with additional 5-HT_2A_-R agonist stimulation after 90 s from distal to proximal (bottom plots). Scale bar, 20 µm

In order to investigate if endogenous 5-HT_2A_-Rs induce similar Ca^2+^ response dynamics in cortical neurons in 5-HT_2A_-R domains as observed for CaMello-5HT_2A_, we engineered a new tool called mloCal-5HT_2A_ (Fig. 4). This construct consists of a GCaMP6m, mCh, the CT of 5-HT_2A_ and a CAAX domain for membrane localization^34^. This tool allows for monitoring of intracellular Ca^2+^ signals in 5-HT_2A_-R domains by activation of endogenous 5-HT_2A_-R without inserting a full-length GPCR into 5-HT_2A_-R domains. Expression of mloCal-5HT_2A_ in HEK tsA201 cells detects ATP-induced oscillating intracellular Ca^2+^ signals at the plasma membrane (Fig. 4a). Expression of mloCal-5HT_2A_ in OTC neurons reveals localization at the plasma membrane, but is also found in intracellular clusters (Fig. 4b and Supplementary Figure 3) as observed for the CaMello-5HT_2A_, exogenously expressed 5-HT_2A_-Rs and endogenous 5-HT_2A_-Rs (Supplementary Figures 1, 3, 4). The intrinsic activity of the OTCs leads to oscillating Ca^2+^ signals throughout the neurons detected by mloCal-5HT_2A_. These Ca^2+^ signals can be induced by the 5-HT_2A_-R agonist TCB-2 (note, we used saturating agonist concentration (20 µM) to activate endogenous 5-HT_2A_-R, since we also used light-intensities and light-pulse durations, which fully activate CaMello and CaMello-5HT_2A_ responses (see Fig. 2)). Blocking intrinsic activity by TTX and CNQX abolishes the oscillating Ca^2+^ signals, while subsequent activation of endogenous 5-HT_2A_-Rs induces Ca^2+^ signals at specific dendritic and somatodendritic domains.

### Receptor-specific Ca^2+^ dynamics and spiking activity in cerebellar Purkinje cells

**Fig. 5:**
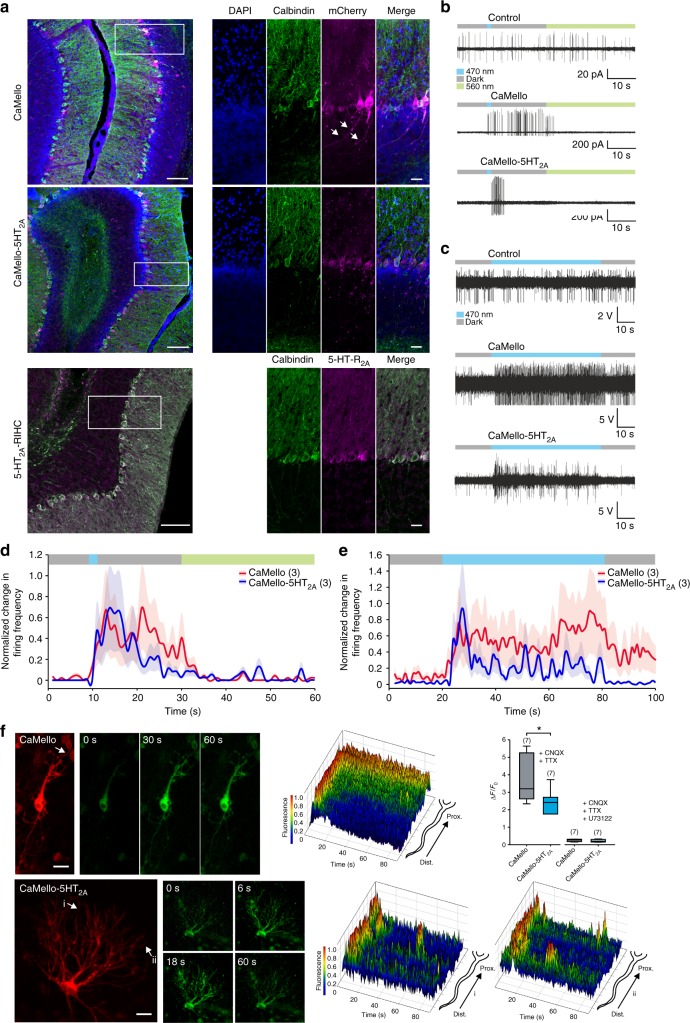
Optogenetic sustained and transient modulation of neuronal firing in the cerebellum in vivo and in vitro combined with visualization of local Ca^2+^ signals. **a** Sagittal images depicting brain sections of the cerebellar cortex expressing CaMello or CaMello-5HT_2A_ compared to the native 5-HT_2A_ receptor (IHC: immunohistochemistry). Scale bar, 100 µm overview, 25 µm zoom. **b** Example traces of light-induced modulation of Purkinje cell (PC) firing in cerebellar slices by CaMello, CaMello-5HT_2A_ or mCherry control. The protocol to control the receptor consisted of an initial 10 s dark phase (gray) followed by a 1 s, 470 nm light pulse (blue) to activate the receptor, followed by an additional dark phase of 19 s and a 30 s, 560 nm light pulse (green) for deactivation of the receptor. **c** Example traces of in vivo optrode PC recordings from anaesthetized mice expressing CaMello, CaMello-5HT_2A_ or mCherry control. The protocol to control the receptor consisted of an initial 10 s dark phase (gray) followed by a 60 s, 470 nm light pulse (blue) and an additional dark phase of 10 s. **d**, **e** Light-induced change in firing frequency for cerebellar slice recordings (**d**) or in vivo optrode recordings (**e**). Change in firing frequency during the indicated protocol (see color bar and **b**, **c**) was normalized for each individual cell for CaMello and CaMello-5HT_2A_ and pooled (mean ± s.e.m.; *n* = 3 animals per group). **f** Time course of light-induced local Ca^2+^ responses in rat cerebellum OTCs. Transfected cells were visualized using the mCherry reporter and Ca^2+^ signals were light-induced (476 + 495 nm) and measured via GCaMP6m monitoring (images). 3D mesh plots of individual neurites (arrow: CaMello; i, ii: CaMello-5HT_2A_) showing normalized local light-induced Ca^2+^ responses during 90 s of illumination from distal to proximal (plots). Comparison of maximal change in induced fluorescence intensity (∆*F*/*F*_0_). Maximal change in ∆*F*/*F*_0_ in the presence of CNQX (1 µM) and TTX (1 µM) or additional PLC antagonist U73122 (10 µM) (box plot; unpaired *t*-test; *n* = 7 individual cells, pooled from four animals per group; *p < 0.05; *p* = 0.031) (box plot). Scale bar, 25 µm

The cerebellar cortex receives innervation of serotonergic neurons and various 5-HTRs are expressed in the cerebellum. Within cerebellar Purkinje cells (PCs), the sole output neurons of the cerebellar cortex, 5-HT_2A_-Rs are endogenously expressed at the soma and dendrites, but not in the axon^35,36^ (Fig. 5a). We next investigated the distribution of CaMello, CaMello-5HT_2A_, and endogenous 5-HT_2A_ and how GPCR activation affects the Ca^2+^ responses and action potential firing of PCs. CaMello distributes throughout the neurons and could be found in dendrites, the soma and the axon. CaMello-5HT_2A_ and endogenous 5-HT_2A_-Rs are excluded from the axon and reveal a more clustered distribution throughout the soma and dendrites. In the presence of CaMello a 1 s blue light pulse induces immediate sustained PC spiking in cerebellar slices (Fig. 5b, c) and in vivo (Fig. 5c, e), which can be switched off with yellow light. In contrast, activation of CaMello-5HT_2A_ in PCs leads to a transient increase in PC spiking in cerebellar slices (Fig. 5b, d) and an oscillation of PC firing in vivo (Fig. 5c, e). The differences in spiking behavior of PCs in vitro and in vivo can be related to the Ca^2+^ response dynamics in OTCs. (Note, the cerebellar cortex and the neuronal networks and connections including PCs are not fully developed when OTCs are prepared at postnatal day 6^37,38^. Therefore the morphology of cerebellar cortical neurons are most likely altered in these cultures and we therefore refer here to PC-like neurons). As also observed in cortical neurons, CaMello activation in PC-like neurons induces a strong sustained Ca^2+^ signal in the soma and proximal dendrites and a transient signal at the distal dendrites (Fig. 5f). In contrast, CaMello-5HT_2A_ activation in PCs leads to a strong, transient activation of the Ca^2+^ signal at the soma, proximal and distal dendrites, which oscillates throughout the soma and the dendritic tree (Fig. 5f). These Ca^2+^ signals are abolished by the PLC antagonist U73122, suggesting that Ca^2+^ responses are activated via the G_q/11_ pathway (Fig. 5f).

### Visually evoked changes of network activity in the visual cortex in vivo

**Fig. 6:**
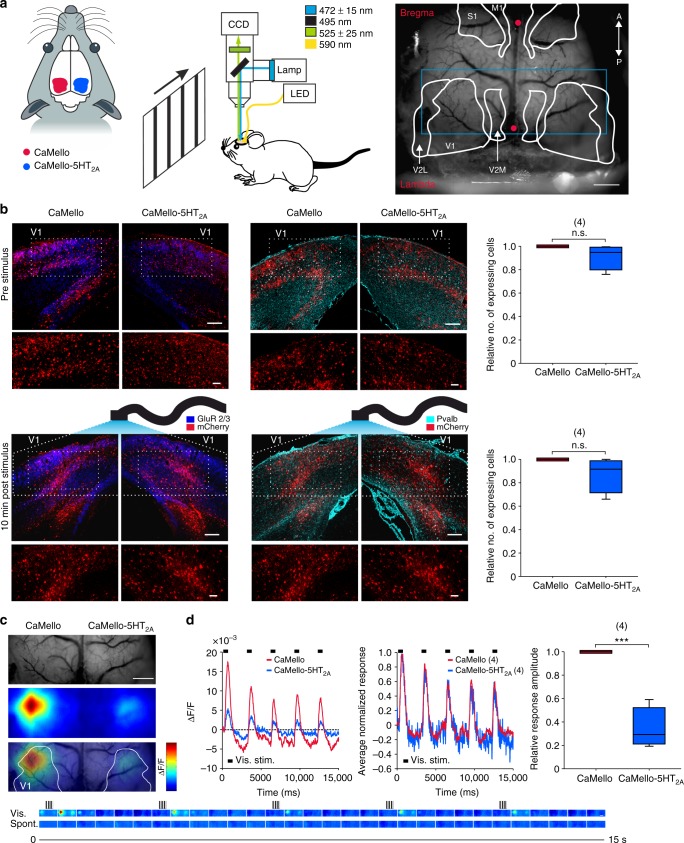
Simultaneous photostimulation and Ca^2+^-imaging of visually evoked responses in the primary visual cortex in vivo. **a** Scheme of the experimental setup. Visual stimuli were presented on a monitor at 20 cm in front of the mouse. Between recording (and photostimulation) sessions, yellow light (590 nm) was used to deactivate the constructs. Mice were anesthetized and head-fixed. Vascular pattern of the cortex overlaid with schematics showing different cortical regions (V1: primary visual; V2L: lateral secondary visual; V2M: medial secondary visual; S1: primary sensory; M1: primary motor; red dots: bregma/lambda; blue rectangle: imaged area, depicted in (**c**)). Scale bar, 1 mm. **b** Coronal images depicting brain sections of the visual cortex expressing CaMello and CaMello-5HT_2A_ for animals before and 10 min after stimulation. Pyramidal neurons were antibody-stained against GluR2/3, while Pvalb+ neurons were stained against parvalbumin (GluR: glutamate receptor; Pvalb: parvalbumin) (images). The relative number of expressing cells in the illuminated area was compared for animals before/after stimulation (box plot; unpaired *t*-test; *n* = 4 animals per group; n.s. = not significant; *p* from top to bottom: 0.079, 0.067) (box plot). Scale bar, 150 µm overview, 50 µm zoom. **c** Depiction of the imaged area. Vascular pattern of the imaged cortical region (top). Activation across V1 and neighboring visual areas after visual stimulation with changes in activity over time shown as relative change in fluorescence (∆*F*/*F*) (middle). Overlay of the two images above (bottom). Image frames show GCaMP6m signals in response to visual stimulation (upper row, vis.) with moving gratings (see icons on top) and spontaneous activity (lower row, spont.). Changes in activity over time are expressed as relative change in fluorescence (∆*F*/*F*). Each frame represents the average fluorescence change across 500 ms of recording. Scale bar, 1 mm. **d** Traces depicting the time course of spatial averages across V1 for CaMello and CaMello-5HT_2A_ in response to visual stimulation, average of 30 trials (left). Comparison of the averaged (*n* = 4 animals) normalized responses of both constructs (middle). Comparison of maximal visual response amplitudes (box plot; unpaired *t*-test; *n* = 4 animals; ***p < 0.001; *p* = 0.000137) (right)

We next investigated the influence of the G_q/11_ induced Ca^2+^ signals during neuronal processing in the primary visual cortex (V1) in vivo using wide-field optical imaging. V1 receives strong serotonergic innervation from the dorsal raphe nucleus and mediates its effect in particular via 5-HT_2A_-Rs expressed in pyramidal cells as well as parvalbumin positive (Pvalb^+^) interneurons^7,12,39^. We compared the effects of G_q/11_ induced Ca^2+^ signals during visual processing between CaMello and CaMello-5HT_2A_, which were mainly expressed in pyramidal neurons (immunostaining reveals colocalization with the endogenous GluR2/3 subunits in pyramidal neurons) and Pvalb^+^ interneurons (immunostaining reveals colocalization with parvalbumin) after viral infection of V1 (Fig. 6b). Prior to and immediately after stimulation (stimulation protocol see below and Fig. 6c (bottom)) the relative number of tool expressing (mCh fluorescent) cells was similar across both hemispheres with no significant differences between both constructs (Fig. 6b). However, we found that 24–72 h post stimulation the relative number of tool expressing cells was significantly reduced for CaMello-5HT_2A_ (Supplementary Figure 6). The reduction in CaMello-5HT_2A_ expressing cells post stimulation may involve GPCR/DNA/virus degradation or long-term intrinsic regulatory mechanisms within the visual cortex. Repetitive presentation of visual stimuli (five times, 200 ms duration) resulted in transient Ca^2+^ responses, which were larger for CaMello in comparison to CaMello-5HT_2A_ and declined in amplitude during repetition of stimuli (Fig. 6c) (Note: repetitive visual stimuli were applied during activation of the Gq pathway via the light-activated GPCR and monitoring intracellular Ca^2+^ levels via GCaMP6m). After normalization of the traces to their individual maxima (Fig. 6d), we found a striking overlap between the curves. The smaller response amplitudes for CaMello-5HT_2A_ could be related to the activity-dependent internalization of the CaMello-5HT_2A_ or/and to suppression of the gain of evoked visual responses mediated by the activation of G_q/11_ signals in 5-HT_2A_ receptor domains.

## Discussion

GPCRs activate a plethora of intracellular signaling cascades^17,40^. Very little information is available about the intracellular activation of these pathways in their native environments. In particular, we do not know which combination of intracellular pathways are spatially and temporally activated and regulated and how these GPCR pathways interact in and between different microdomains. Therefore, new tools have to be developed to decode the activation of GPCR signaling cascades in their specific subcellular environment.

One of the main restrictions to understand the role of intracellular signaling cascades in the brain is the limitation of tools to resolve these signaling cascades in a spatial and temporal manner in the living brain. Various FRET-based sensors have been developed and applied in particular to monitor changes in intracellular cAMP levels, ERK or RAC1 activity^41^. The tool of choice for monitoring changes in intracellular Ca^2+^ levels is currently GCaMP6, which is based on a circularly permuted GFP^19^. Various Ca^2+^ sensors including GCaMPs have been developed^42^, but only a few studies analyzed Ca^2+^ signaling in protein-specific signaling domains such as adenylyl cyclase domains^43^, voltage gated Ca^2+^ channel domains^44^, and 5-HT_6_ receptor domains^45^ (for review see ref. ^42^). The main limitation to these studies is that they were performed in cultured cell lines and not in a native environment like the brain.

We now developed CaMello and CaMello-XR to precisely control and monitor changes in G_q/11_/PLC-mediated intracellular Ca^2+^ levels and to correlate the intracellular Ca^2+^ response with the cellular trafficking of the GPCR. We therefore first identified an insertion site for a red fluorescent protein (mCh). As previously reported^20^, we found that replacing the intracellular loop III of melanopsin with mCh does not interfere with the activation and deactivation kinetics of mouse melanopsin (Figs 1 and 2). Second, we fused GCaMP6m to the CT of moMo, which allows for monitoring Ca^2+^ signals at the plasma membrane. Third, we C-terminally tagged CaMello with the CT of the 5-HT_2A_ receptor, which targets CaMello to the domain, where endogenous 5-HT_2A_-Rs reside (Figs 1, 3–5 and Supplementary Figure 4). This optogenetic tool now allows the correlation of GPCR trafficking within its subcellular microdomain and simultaneous activation and visualization of the G_q/11_/PLC-mediated increases in intracellular Ca^2+^ levels. This method might be applicable to every GPCR of choice in order to decipher Ca^2+^ signals in GPCR-specific receptor domains in the brain. However, this has to be established and verified for every target GPCR separately.

GPCR-mediated pathway activation and pathway selectivity is very complex. GPCRs often have multiple effectors, activate various signaling pathways, which can be dependent on the ligand concentration. In addition, signaling may depend on the GPCR localization in and within subcellular structures (i.e., plasma membrane, endosomes or presynaptic terminal or postsynaptic spine) and the heterodimerization between different GPCR subtypes^46,47^. Activation of neuronal 5-HT_2A_-Rs have also been described to be pleiotropic and involve G protein-dependent, ligand-dependent, and ligand-independent effects leading to the activation of, for example, PLC/PKC, ERK or β-arrestin2/phosophinositide 3 kinase/Src/Akt signaling cascades^48^. The most detailedly analyzed pathway is currently the activation of the Gq-PLC pathway leading to an increase in intracellular Ca^2+^ levels^48,49^. In this study, we concentrated on creating tools to specifically monitor PLC-mediated changes in intracellular Ca^2+^ levels throughout neurons and within or at least in close proximity to 5-HT_2A_-R domains. While 5-HT_2A_-Rs are mainly localized to somatodendritic domains they can also be found in axon terminals^48^. Therefore, in our study we cannot exclude the contribution of axonal Ca^2+^ signals detected by CaMello, CaMello-5HT_2A_, and mloCal-5HT_2A_.

Expression of CaMello and CaMello-5HT_2A_ in cerebellar PCs and cortical neurons revealed differences in the amplitude and spatial activation pattern between 5-HT_2A_-targeted and non-targeted CaMello. A global, sustained Ca^2+^ signal is induced by CaMello, which reveals a high expression level at the soma and proximal dendrites. The sustained increase in somatic Ca^2+^ levels are correlated with a sustained increase in firing in PCs. In contrast, transient Ca^2+^ puffs and waves are induced by CaMello-5HT_2A_, which often spread from the distal dendrites to the soma (Supplementary Movies 3–6). The occurrence of the transient Ca^2+^ puffs seem to be randomly distributed over the dendritic tree, with each individual dendrite apparently acting independently^50^. The occurrence of randomly distributed, transient Ca^2+^ puffs could also be observed with mloCal-5HT_2A_ when endogenous 5-HT_2A_-Rs were activated (Fig. 4). This tool allows for visualization of Ca^2+^ responses in 5-HT_2A_-R domains without inserting full-length GPCRs into the receptor domain targeted by the CT of 5-HT_2A_-R.

According to the transient Ca^2+^ signals induced by CaMello-5HT_2A_, AP firing in cerebellar PCs is transiently increased in vitro and oscillates in vivo. Interestingly, we also observed different response amplitudes for CaMello vs. CaMello-5HT_2A_ after visually evoked neuronal activity in V1. The differences in response amplitude during visual stimulation suggests that activation of the G_q/11_ pathway in 5-HT_2A_ receptor domains specifically contributes to visual processing via suppression of evoked activity that may have a divisive scaling effect on the visual response amplitudes^51^.

The subcellular targets of the modulation for 5-HT_2A_ are not very well understood in cortical pyramidal neurons and cerebellar PCs, but most likely involve changes in intracellular Ca^2+^ levels leading to increased excitability and plasticity effects^13,18,35^. The increase in intracellular Ca^2+^ can either directly contribute to the depolarization of the cell membrane or can modulate other signaling cascades leading to decreases in slow afterhyperpolarization, PKC-mediated reduction of Na^+^ currents, potentiation of LTP and NMDA receptor regulation involving the ERK pathway^7,13,52,53^.

The experiments suggest that functionally different 5-HT_2A_ microdomains exist within neurons, which may determine the modulation of different cellular targets and may contribute to supralinear Ca^2+^ accumulation and coincidence detection, which has been demonstrated for group 1 mGluRs^54^. For example, repetitive synaptic stimulation induces an mGluR-dependent activation of IP_3_-dependent Ca^2+^ transients, which spread as a somatodendritic wave in CA1 pyramidal hippocampal neurons^55^. In contrast, in cerebellar PCs mGluR1-dependent intracellular Ca^2+^ transients are restricted to postsynaptic microdomains which range from individual spines to small spinodendritic compartments^56^. Thus, correlating GPCR-dependent activation with physiological function depends on the exact localization of the GPCR within the microdomain and activation of microdomain-specific signaling pathways. CaMello-XRs now allow specific control and visualization of these pathways within the microdomains.

Regulation and understanding of GPCR trafficking are crucial for determining the signal response amplitudes and kinetics of an activated signaling pathway. These signal responses are GPCR-specific and depend on intracellular interacting proteins such as β-arrestin and PDZ domain containing proteins. They occur not only at the plasma membrane but also in subcellular domains such as endosomes and the Golgi apparatus^57^. In respect to 5-HT_1_-Rs and 5-HT_2_-Rs, β-arrestin mediates the agonist-induced internalization of 5-HT_1A_ and an agonist-independent internalization of 5-HT_2C_-Rs^58^. In contrast, the trafficking of 5-HT_2A_-Rs is independent of β-arrestin but dependent on dynamin^31^, which requires the CT of 5-HT_2A_ (see Figs 1 and 4). In fact, using the CT of 5-HT_2A_-R is sufficient to localize CaMello-5HT_2A_ to early and late endosomes (Figs 1 and 3). 5-HT_2C_-Rs both bind PDZ domain containing proteins, which influence subcellular localization and internalization in a receptor- and binding protein-specific manner^59^. For example, PSD-95 prevents the internalization of 5-HT_2A_-Rs^10^, while it induces the agonist-dependent and constitutive endocytosis of 5-HT_2C_-Rs. Many of the protein domains necessary for anchoring and regulation of GPCR function are located in the CT^59^. For example, 5-HT_2A_-Rs contain a PDZ-binding domain at the C-terminal end of the CT^9^. The 5-HT_2A_ CT also binds calmodulin, which prevents PKC phosphorylation and receptor desensitization^60^. Thus, the signaling response depends on the subcellular localization and the interacting proteins within a microdomain of the GPCR and might be cell-type- and microdomain-specific. Previous studies demonstrated that using the CT of 5-HT_1A_-R, 5-HT_1B_-R, and 5-HT_2C_-R is sufficient to target optogenetic tools such as vertebrate opsins to the specific 5-HT receptor domains to control and visualize the signaling responses in a receptor-specific manner^21–24^. Thus, CaMello and CaMello-XR will help to elucidate the subcellular signal activation for specific 5-HT receptors and can most likely be extended to other GPCRs.

GPCRs activating the G_q/11_ pathway have been identified as important drug targets for various neurological diseases. For example changes in the function of group I mGluRs (i.e., mGluR1 and mGluR5) have been implicated in cerebellar ataxias, extrapyramidal motor dysfunctions, fear and anxiety, mood disorders, epilepsy, and pain^61^. Malfunction of 5-HT_2_ receptors have been implicated in particular in neuropsychiatric disorders such as bipolar and anxiety disorders, depression, schizophrenia and migraine as well as cardiovascular disorders^62^. Since changes in the activity and function of these receptors, including changes in the targeting, expression levels, internalization, and kinetics of the signaling responses lead to the deleterious changes in brain function, it will be important to monitor the trafficking and signal kinetics and amplitudes directly at the GPCR level. To our knowledge, this method is the first, which allows for precise monitoring, visualization and analysis of G_q/11_/PLCβ activation in neurons and has the potential to detect changes in intracellular Ca^2+^ signaling in subcellular microdomains of GPCRs underlying disease phenotypes.

## Methods

### Generation of plasmid constructs

The long isoform of mouse melanopsin (mOpn4L or moMo, GenBank accession number: NM_013887.2) was used as the backbone construct for the generation of CaMello and CaMello-5HT_2A_. To construct AAV-expression vectors and allow for the necessary large packaging capacity, the pAAV-CW3SL-EGFP vector (GenBank accession number: KJ411916.2) was used as the backbone plasmid for all constructs^63^. Each element was PCR amplified with 16 bp overhangs and inserted into the backbone via AQUA Cloning, replacing the DNA sequences between the inverted terminal repeats (ITRs) and exchanging the CamKIIα promoter with CMV^64^. For the CaMello and CaMello-5HT_2A_ constructs, the mCh reporter was inserted into the third intracellular loop (il3) of mOpn4L replacing il3 (see refs. ^65,66^), and the GCaMP6m reporter was added C-terminally^19^. To generate the CaMello-5HT_2A_ construct the C-terminus (amino acids K385-V471) of the human 5-hydroxytryptamine receptor 2A (HTR2A, GenBank accession number: NM_000621.4) was added C-terminally to the CaMello construct. The 5-HT_2A_ control plasmids were generated accordingly, via C-terminal addition of mCh or GCaMP6m to the full-length receptor. The endosomal markers mCitrine-Rab5a and mCitrine-Rab7a were gifts from Michael Davidson (Addgene plasmids # 56566 and # 56567) and the trans-Golgi network marker GALT-mCitrine (beta-1,4-galactosyltransferase 1 with C-terminal mCitrine) as well as the postsynaptic marker PSD-95 eGFP (postsynaptic density protein 95 with C-terminal eGFP) were generated in the lab. To generate the mloCal-5HT_2A_ construct N-terminal GCaMP6m was fused to mCherry followed by the 5-HT_2A_-R C-terminus via short linker sequences and lipid anchored to the cellular membrane through addition of a C-terminal isoprenylated CAAX box (C-terminal region of the human GTPase KRAS isoform B, GenBank accession number: XM_011520653.3, amino acid sequence: KEKMSKDGKKKKKKSKTKCVIM)^67^.

### Cell culture

Human embryonic kidney (HEK) tsA201 cells and HEK GIRK 1/2 cells (HEK293 cells stably expressing GIRK1/2 subunits, kindly provided by Dr. A. Tinker UCL London, GB) were maintained at 37 °C in Dulbecco’s modified Eagle’s medium (DMEM), 4.5 g l^−1^ D-glucose, supplemented with 10% fetal bovine serum (Gibco) and penicillin/streptomycin in a humidified incubator under 5% CO_2_. Growth medium of stable cell lines was supplemented with G418 (5 mg/ml). Cells were cultured on 35 mm glass bottom dishes (for imaging) or plastic bottom dishes (for electrophysiology). Cells were transfected with FuGENE® HD (Promega) according to the manufacturer’s protocol and incubated for 18–24 h before recordings. For localization and trafficking experiments cells were additionally serum-starved for 12 h prior to experiments. For opsin experiments retinal (9-*cis* or all-*trans* with no difference detectable, see ref. ^20^) was added to a final medium concentration of 1 µM. To image Ca^2+^ signals in HEK tsA201 cells via GCaMP6m, cells were transiently transfected with CaMello, CaMello-5HT_2A_ or mloCal-5HT_2A_. Cells were seeded into poly L-lysine coated 35 mm glass bottom dishes, transfected at 70% confluency with equal amounts of plasmid DNA and used the next day. Ca^2+^ imaging was performed at an inverted Leica TCS SP5 confocal laser-scanning microscope, (Leica DMI6000 B, Wetzlar, Germany) interfaced to a personal computer, running Leica Application Suite Advanced Fluorescence software (LAS AF 2.6). A 20X/0.7NA (Ca^2+^ imaging) and a 40X1.1/NA (receptor internalization) objective were used to acquire timelapse images (512 × 512 pixels with 0.5 s interval for live cell imaging). Cells were visualized via mCh fluorescence with the 561 nm laser, concomitantly deactivating CaMello or CaMello-5HT_2A_. CaMello and CaMello-5HT_2A_ were activated with the 476 and 495 nm argon laser lines. For CaMello-5HT_2A_ control experiments Dynasore (50 µM, Sigma-Aldrich) was applied 30 min before measuring. For mloCal-5HT_2A_ experiments 10 µM ATP was bath applied to induce Ca^2+^ responses. Emission spectrum of GCaMP6m was monitored between 500 and 550 nm. Fluorescence intensity of the GCaMP6m signal was measured over time for individual cells, normalized and scaled to the maximal response amplitude.

For localization experiments in HEK cells CaMello-5HT_2A_ or the 5-HT_2A_ receptor mCherry construct were coexpressed with mCitrine-Rab5a, mCitrine-Rab7a or GALT-mCitrine and stimulated with high/low intensity 476 nm light, TCB-2 (20 µM) or 5-HT (1 µM) for 5 min and sequential images of the mCherry and mCitrine fluorescence were acquired. Captured images were transferred into ImageJ software (1.47 v; NIH) and Pearson’s correlation coefficient was calculated for individual cells with the ImageJ Coloc 2 plugin.

For internalization experiments Dynasore (50 µM; obtained from Sigma-Aldrich) was applied 30 min before measuring and TCB-2 (20 µM; obtained from Tocris Bioscience) was bath applied directly before measuring. Membrane-localized mCh fluorescence intensity was measured over time for individual cells, normalized, scaled to the maximal response amplitude and monitored between 570 and 650 nm with and without simultaneous activation of CaMello/CaMello-5HT_2A_ (via 476 nm argon laser stimulation) or stimulation of the 5-HT_2A_-R (via bath application of the 5-HT_2A_-R agonist TCB-2). To determine activation-dependent receptor internalization, pooled and averaged data with and without stimulation were subtracted from each other. For control experiments the dynamin inhibitor Dynasore was applied to block receptor internalization. Captured images were transferred into ImageJ software (1.47 v; NIH) and analyzed with the time series analyzer V3 plugin.

### In vitro electrophysiology

For GIRK channel recordings light-sensitive GPCRs were expressed in HEK GIRK 1/2 cells (see above). Cells were cultured on 35 mm dishes and recorded in dark room conditions after transfection. GIRK-mediated K^+^-currents were measured and analyzed as described in the following (see also ref. ^68^). The external solution was as follows: 20 mM NaCl, 120 mM KCl, 2 mM CaCl_2_, 1 mM MgCl_2_, 10 mM HEPES-KOH, pH 7.3 (KOH). Patch pipettes (2–5 MΩ) were filled with internal solution: 100 mM potassium aspartate, 40 mM KCl, 5 mM MgATP, 10 mM HEPES-KOH, 5 mM NaCl, 2 mM EGTA, 2 mM MgCl_2_, 0.01 mM GTP, pH 7.3 (KOH). Cells were recorded in external solution containing 1 µM 9-*cis* retinal (Sigma). Experiments were conducted with an inverted microscope (Axiovert, ZEISS) and patch pipettes were controlled with a multi-micromanipulator (MPC-325, SUTTER INSTRUMENT). Transfected cells were visualized and CaMello-XRs were manipulated with a monochromator system (Polychrome V, TILL Photonics). Whole-cell patch clamp recordings of HEK cells were performed, digitized at 10 kHz and filtered with an EPC10 USB amplifier (HEKA). Series resistances were partially compensated between 70 and 90%. The PatchMaster software (HEKA) was used for monochromator and voltage controls as well as data acquisition, and off-line analysis was made with Igor Pro 6.0 software (Wavemetrics). The GCaMP6m excitation spectrum was measured via activating CaMello for 1 s with a light pulse of 470 nm and subsequently exciting the (CaMello-) GCaMP6m between 390–520 nm in 10 nm steps while recording the corresponding emission between 530 and 550 nm with an sCMOS Camera (PRIME, Photometrics) attached to a dual-channel simultaneous-imaging system (DVΛ, Photometrics) with appropriate filter settings.

### Live vertebrate studies

Long-Evans RjOrl:LE rats (strain code #006, outbred) were obtained from Charles River and C57BL/6J (stock #000664) mice were obtained from the Jackson Laboratory. Mice were bred and raised in the animal facility of the Department of Zoology and Neurobiology and housed individually on an 8 a.m. to 8 p.m. light/dark schedule. Rats were bred and raised in the departmental animal facility and housed pairwise on an 8 a.m. to 8 p.m. light/dark schedule. All experiments were approved by the Institutional Animal Research Facility (Ruhr-University Bochum) and the local government ethics committee (LANUV NRW, (Landesamt für Umwelt, Natur und Verbraucherschutz Nordrhein Westfalen, Düsseldorf)). The age and species used for each experiment is specified below within the corresponding method description.

### AAV2 virus production and stereotactic virus injection

Recombinant AAV stocks of serotype 9 were produced via the triple-transfection method^69^ and purified using chloroform. In short, HEK293T cells were transfected with the vector of interest, the serotype plasmid and helper plasmid using polyethylenimine. Seventy-two hours after transfection, cells were harvested via low-speed centrifugation. Cells were resuspended in lysis buffer (150 mM NaCl, Tris Cl pH 8.5), freeze-thawed 5–7 times and incubated with DNaseI plus MgCl_2_ at 37 °C for 30 min. PEG-8000 (10% final w/v) was added to the supernatant and the mixture incubated for 2 h at 4 °C. After centrifugation at 3700 × g for 20 min at 4 °C, the PEG-precipitated pellet was resuspended in the clarified cell lysate. For purification, the resuspension was incubated with PEG-8000 for ≥ 1 h at 4 °C, centrifuged (3700 × g, 4 °C, 20 min), and the pellet resuspended in 50 mM HEPES buffer. Afterward, room-temperature chloroform (1:1 volume) was added, the mixture vortexed and then spun down at 370 × g at RT for 5 min. The aqueous phase was collected, filtered using a syringe filter (0.22 µm) and concentrated using PEG-8000. The concentrated AAV was resuspended in 1x PBS with 0.001% pluronic F68, aliquoted and stored at −80 °C.

For cerebellar in vivo and in vitro electrophysiological experiments adult wild-type male (C57Bl/6J) mice aged 1–3 months were anesthetized with an initial dose of isoflurane and placed into a stereotaxic frame. Body temperature was controlled with a heating pad and anesthesia was maintained with 1.8–2.0% isoflurane for the entire session. To prevent corneal drying during surgery the eyes were coated with a moisturizing balm. Animals were sheared from the top of the head and the skin was opened with a sagittal incision along the midline. A burr hole was drilled for virus delivery above the cerebellar vermis (stereotactic coordinates from bregma: −6.5–7 mm anteroposterior (A/P); 0 mm mediolateral (M/L); −2000 dorsoventral (D/V)). A customized glass pipette (tip diameter about 10 µm) attached to a 10 ml syringe was used to deliver AAV solution containing either CaMello or CaMello-5HT_2A_ via pressure injection in 200 µm steps starting from −2000 µm. After the surgery animals received subcutaneous injections of carprofen (2 mg/kg) for analgesia. Animals were placed individually into their homecages and allowed to recover for at least 7–14 days before performing electrophysiological experiments.

For cortical in vivo simultaneous photostimulation and Ca^2+^ imaging experiments wild-type male (C57Bl/6J) were anesthetized with isoflurane in oxygen (3% induction, 1.0% maintenance), while kept on a heating blanket at 37 °C. Additionally, subcutaneous injection of 0.5 ml saline solution containing 2 µg/ml buprenorphine and 3 µg/ml atropine in saline was administered during the surgery as analgesic agents. Before the incision of the scalp, 2% Lidocaine was applied on the scalp to provide additional local anesthesia. The skull was thinned until surface blood vessels became clearly visible. Three small craniotomies (4.2 mm posterior, 2.5 mm lateral; 3.5 mm posterior, 2 mm lateral and 3.3 mm posterior, 2.7 mm lateral to Bregma) were performed above the primary visual cortex (V1) of both hemispheres. The mice were injected in V1 of each hemisphere with 2–3 µl virus containing either CaMello or CaMello-5HT_2A_. Viral solution containing CaMello or CaMello-5HT_2A_ was delivered via small pressure injections (at −0.8, −0.55, and −0.3 mm depths, 10 min apart). The thinned skull was covered with transparent dental cement and nail polish. A small head holder was cemented to the skull to allow head fixation and a chronic imaging window and the skull was covered with Kwik-Cast (WPI, Sarasota, FL, USA) to avoid exposure of CaMello/CaMello-5HT_2A_ to light. The animals were allowed to express the virus and recover for at least two weeks before the Ca^2+^ imaging experiments.

### Brain slice recordings

Parasagittal cerebellar slices were cut from cerebellums of mice 14 days after AAV9 injection and recordings were performed as follows. Briefly, mice were anesthetized with isoflurane and decapitated. The cerebellum was sliced in ice-cold artificial cerebrospinal fluid containing 125 mM NaCl, 2.5 mM KCl, 2 mM CaCl_2_, 1 mM MgSO_4_, 1.25 mM NaH_2_PO_4_, 26 mM NaHCO_3_, and 20 mM glucose bubbled with 95% O_2_ and 5% CO_2_ using a vibratome (VT1000S, Leica) and the slices were then stored for at least 1 h at 37 °C in this solution. Fluorescent mCh-positive cells were visually identified under an upright microscope (DMLFSA, Leica) equipped with a monochromator system (Polychrome IV, TILL Photonics) flashing excitation light (light intensity, 1.8 mW/mm^2^ for 470 nm and 1.2 mW/mm² for 560 nm). Cell-attached recordings were made at 37 °C in the dark except for using infrared light to target the cell. Slices were preincubated at least 20 min and continuously perfused with the external solution including 25 µM all-trans-retinal, 0.025% (±)-α-tocopherol (Sigma), 0.2% essentially fatty acid free albumin from bovine serum (Sigma), and 0.1% dimethyl sulfoxide. Patch pipettes (4–8 MΩs) were filled with an internal solution with the composition 125 mM potassium gluconate, 4 mM NaCl, 2 mM MgCl_2_, 10 mM HEPES, 0.2 mM EGTA, 4 mM Mg-ATP, 0.4 mM Na-GTP, and 10 mM Tris-phosphocreatine, pH 7.3 (KOH). The holding potential for current recordings was −60 mV unless stated otherwise. Membrane currents and voltages were recorded with an EPC10/2 amplifier (HEKA). The signals were filtered at 3 kHz and digitized at 10 kHz. One trial lasted 60 s, including 10 s baseline recordings in darkness, 1 s light stimulation with 470 nm to activate CaMello (−5HT_2A_), followed by 19 s recordings in darkness and 30 s light stimulation with 560 nm to deactivate CaMello (−5HT_2A_), with five trials being recorded per cell. The PatchMaster software (HEKA) was used for the controls of voltage and data acquisition, and off-line analysis was made with Igor Pro 6.0 software (Wavemetrics).

### In vivo extracellular recordings and optical stimulation

For extracellular in vivo recordings, anaesthetized mice were placed into a stereotactic frame 14 days after AAV9 injection. Optrodes consisted of an optical fiber with 200 µm diameter (Thorlabs, FT200-UMT) fused to a customized glass-coated tungsten recording electrode (2–4 MΩ). Optrodes were coupled to a blue LED module (465 nm Plex Bright LED, Plexon) for light delivery. Light intensity at the tip of the optrode was 1–3 mW/mm^2^. Single- and multi-unit potentials were amplified and filtered (Gain 10 kHz; 300 Hz–10 kHz band-pass; A-M Systems, model 1800). After noise elimination (50/60 Hz Noise Eliminator, Quest Scientific) potentials were stored with a sampling rate of 20 kHz using a 1401 Power mk interface (CED) and analyzed offline using Spike2 software. One trial lasted 100 s, including 20 s baseline recordings, 60 s light stimulation, followed by 20 s additional baseline recordings with five trials being recorded per cell. Individual spikes were sorted offline either by action potential shape or individual threshold. mCh expressing cells were used as control. Single- and multi-units were exported as Matlab files. Data analysis was done offline by a customized Matlab program.

### Preparation of rat organotypic cultures (OTCs)

OTCs from rat visual cortex were prepared as described^33,70^. Neonatal rats (pigmented Long Evans from own breeding) were decapitated and visual cortex blocks were quickly recovered. Subsequently, the cortex was sliced into 350 µm sagittal sections using a MCIlwain tissue chopper (Ted Pella, Redding, CA, USA). Cortical slices were mounted on coverslips with a plasma/thrombine coagulate and cultivated in medium containing: 10% adult horse serum, 50% Eagle’s basal medium, 25% HBSS, 1 mM L-glutamine (all from Life Technologies, Karlsruhe, Germany), 8 mM D-glucose (Merck, Darmstadt, Germany), supplemented with NeuroCult^TM^ SM1 at 200 µL/20 mL medium (STEMCELL Technologies Germany GmbH, Köln, Germany). After two days in vitro, 10 µl of mitosis inhibitor solution (1 mM of uridine, cytosine-ß-D-arabino-furanosid and 5-fluordeoxyuridine; each stock 1 mM, all from Sigma) was added to the medium for 24 h to prevent excessive glial growth. Medium change was carried out two times weekly. For cerebellar OTCs, rats were prepared at P6 and sagittal slices at the level of the vermis were cultivated as described above.

### Biolistic and viral transfection of rat OTCs

Transfection of OTCs was carried out according to published procedures^33,70^. In brief, gold nanoparticles (Biorad, Munich, Germany) with 1 µm diameter were loaded with 10 µg plasmid DNA per construct. OTCs were transfected at the below indicated number of days in vitro with a hand-held gene gun (Biorad, Munich, Germany) and 130 psi helium blast pressure. Subsequently, OTCs were given at least three days for recovery and overexpression before imaging was carried out.

The biolistic transfection of cerebellar OTCs turned out to be too inefficient, in particular with regards to obtain Purkinje cell transfectants. Therefore, we used a viral approach to transfect Purkinje cells in the OTCs using our CaMello and CaMello-5HT_2A_ AAV9. Briefly, 2 µl of virus solution was added to 700 µl culture medium and given at least five days for infection and overexpression before Ca^2+^ imaging.

### OTC recordings

For the Ca^2+^ signal recordings single neurons transfected at DIV5 with CaMello, CaMello-5HT_2A_, mloCal-5HT_2A_ or the 5-HT_2A_ receptor GCaMP6m construct were given three days for overexpression. At the day of recording, the culture medium was replaced by prewarmed (37 °C) carbogenated ACSF (containing in mM: 125 NaCl, 5 KCl, 2 CaCl_2_, 1 MgSO_4_, 25 NaHCO_3_, 1.25 NaH_2_PO_4_, 25 glucose, pH 7.4) with multiple washing steps. After recovery (for at least 1 h in the incubator), a single culture was transferred into a custom built heated (30 ± 3 °C) recording chamber mounted to a Leica TCS SP5 confocal laser-scanning microscope, (Leica DMI6000 B, Wetzlar, Germany) interfaced to a personal computer, running Leica Application Suite Advanced Fluorescence software (LAS AF 2.6). Cells were visualized via mCh fluorescence for CaMello, CaMello-5HT_2A_, and mloCal-5HT_2A_ (561 nm laser, concomitantly deactivating the opsin based constructs) or the GCaMP6m fluorescence for the 5-HT_2A_-R GCaMP6m construct (476 and 495 nm laser). For the Ca^2+^ signal recordings CaMello and CaMello-5HT_2A_ were activated with the 476 and 495 nm argon laser lines whereas the 5-HT_2A_-R was activated via bath application of the 5-HT_2A_-R agonist TCB-2 (20 µM) for 90 s. For mloCal-5HT_2A_ experiments the GCaMP6m signal was monitored for 280 s and TCB-2 (20 µM) was bath applied after the indicated time periods. A 40X1.1/NA objective was used to acquire timelapse images (512 × 512 pixels with 0.5 s interval). Emission spectrum of GCaMP6m was monitored between 500 and 550 nm. Captured images were transferred into ImageJ software (1.47 v; NIH) and the Simple Neurite Tracer plugin was used to trace individual neurites of each neuron analyzed. The length of each dendrite analyzed was normalized from 0 to 1.0 by piecewise linear interpolation to normalize the line profile distance. 3D surface plots for the quantitative analysis of local Ca^2+^ signals and for the quantitave analysis of localization dependent mCherry fluorescence were created with ImageJ. To quantitatively analyze the mCherry fluorescence intensity for each construct and to evaluate the relative membrane proximity, close-up confocal z-section images were used to reconstruct complete somata. Depending on the relative fluorescence intensity changes nucleus, cytoplasm and membrane borders were defined for each individual cell. The membrane to cytoplasm proximity ratio was calculated for each individual cell via measuring the average mCherry fluorescence intensity across the membrane and in the cytoplasm for each z-section, excluding nucleic areas. 3D mesh plots were created with Sigmaplot by plotting recording time against normalized fluorescence intensity against normalized distance from distal to proximal. To compare the maximal change in induced fluorescence intensity the peak in normalized fluorescence intensity for each individual cell was pooled. All experiments, except the indicated mloCal-5HT_2A_ experiments without addition of blockers, were conducted in the presence of CNQX 1 µM (Tocris Bioscience) and TTX 1 µM (Tocris Bioscience). For CaMello-5HT_2A_ control experiments the dynamin inhibitor Dynasore (50 µM, Sigma-Aldrich) was added to the culture medium 4 h before measuring to block receptor internalization. For measuring the pathway specificity, the PLC antagonist U73122 10 µM (Biomol) was added to the recording chamber 30 min before the recording.

To analyze the differential expression pattern of each construct and compare it to the complete 5-HT_2A_-R, the normalized fluorescence of the longest dendrite was plotted against normalized dendritic length for CaMello, CaMello-5HT_2A_, and the 5-HT_2A_-R mCh construct^23^. Z-stack images were acquired to image the entire cell and displayed as a projected image. For quantification of relative fluorescence intensity, imaging parameters were adjusted so that pixel intensity within neurites did not saturate. The Simple Neurite Tracer plugin for ImageJ was used to trace the longest dendrite of each neuron analyzed. Dendrites were identified by mCh fluorescence. Fluorescence intensity was normalized to maximal intensity of each dendrite. The length of each dendrite analyzed was normalized from 0 to 1.0 by piecewise linear interpolation to normalize the line profile distance. Interpolated data were pooled, and the mean ± s.e.m. was plotted against normalized distance. Coexpression and antibody staining experiments as described below under histology were performed accordingly.

### In vivo photostimulation and Ca^2+^ imaging of visual responses

Simultaneous wide-field imaging of fluorescent signals of CaMello and CaMello-5HT_2A_ in V1 was performed using the Imager 3001 (Optical Imaging Inc, Mountainside, NY, USA) 14 days after AAV9 injection. The camera was focused ~300 µm below the cortical surface. Cortical images were recorded at 100 Hz frame rate. For simultaneous Ca^2+^ imaging and photostimulation, CaMello/CaMello-5HT_2A_ was excited and the opsin was activated via epi-illumination with 472 ± 15 nm (~1 mW) for 15 s. Fluorescent signals were passed through a high-pass dichroic mirror at 495 nm and band-pass filtered at 525 ± 25 nm. Between trials, the opsin was deactivated with 590 nm LED light (Plexon Inc, Dallas, TX, USA) for 30 s (~1 mW at the tip of a 0.37 NA fiber).

Vertical square-wave gratings (0.037 cycles/deg) moving at 2 cycles/s were presented on a monitor (refresh rate 100 Hz) placed at a distance of 20 cm in front of the mouse. Eyes were covered with semipermeable zero power contact lenses to prevent them from drying out or corneal edema. Each experiment consisted of 10–30 trials. Each trial comprised two different conditions, repeated twice, presented in pseudorandom order: a blank condition, during which a uniform isoluminant gray screen (53 cd/m²) was shown, and a visually evoked condition, during which moving gratings were presented five times with a duration of 200 ms at an interval of 3 s (during which the blank was presented).

The raw data was processed in two steps. First, to remove the differences in illumination across different pixels, divisive normalization of all pixels was performed (frame zero division). Second, the average of all blanks was subtracted from all traces to remove heart-beat and breathing-related signals and to obtain the normalized fluorescence (Δ*F*/*F*).

### Histology

Mice were deeply anesthetized by an overdosed i.p. injection of ketamine and perfused transcardially with 1x phosphate-buffered saline (PBS) followed by 4% paraformaldehyde (PFA) in PBS. Brains were removed and post-fixed in PFA overnight at 4 °C ensued by tissue immersion in 30% sucrose (w/v) overnight at 4 °C for cryoprotection. For cortical sections coronal brain slices about 30 µm thickness were cut at the injection level using a Cryostat (Leica CM3050 S). The free-floating slices got rinsed (3 × 10 min) with 1x tris-buffered saline (TBS) before incubation in blocking solution consisting of 3% normal donkey serum (NDS) in 0.3% Triton-X-100 for 1 h at room temperature. The slices got incubated overnight at 4 °C in primary antibody solutions containing 3% NDS in 0.3% Triton-X-100 and the antibodies rabbit anti-GluR 2/3 (1:300, 07-598, Merck) or mouse anti-PV (1:1000, P3088, Sigma-Aldrich). After washing (3 × 10 min) with TBS, the sections were incubated for 1 h at room temperature in corresponding secondary antibody solutions consisting of 3% NDS in 0.3% Triton-X-100 and the antibodies goat anti-rabbit Cy5 (1:500, 711-175-152, Jackson ImmunoResearch) or donkey anti-mouse DyLight 650 (1:500, SA5-10169, Thermo Fisher). Right after rinsing (3 × 10 min) with TBS, sections were transferred on Superfrost Plus Microscope Slides and coverslipped with Roti®-Mount FluorCare (Carl Roth). Sequential z-stacks were made for each section and transferred to ImageJ software (1.47 v; NIH) for processing and image overlay. For cell counting experiments the same area in the potentially stimulated region of the visual cortex was chosen for stimulated (10 min and > 24 h post-stimulation) and unstimulated control animals. For each coronal slice section across both hemispheres the number of positively expressing cells (mCh) was counted for CaMello and CaMello-5HT_2A_ and normalized to the maximal number of expressing cells. Normalized section cell counts were pooled for each group.

For cerebellar sections the same procedure was followed with the following exceptions: Brain slices were cut sagitally and coverslipped with additional DAPI for the CaMello and CaMello-5HT_2A_ sections and the primary antibody used was mouse anti-Calbindin (1:500, MO19000, Neuromics). Additionally, an antibody staining of uninjected control mice against the native 5-HT_2A_-R was done with the primary antibody goat anti-SR-2A (1:200, SC-15074, Santa Cruz) and the secondary antibody donkey anti-goat Alexa 633 (1:500, A-21082, Thermo Fisher).

For cortical OTC experiments to analyze the differential expression pattern of each construct OTCs were transfected at DIV5 with CaMello, CaMello-5HT_2A_ or the 5-HT_2A_-R mCh construct, respectively. Overexpression was allowed for three days prior to fixation with warm 4% PFA (in 0.1 M phosphate buffer, pH 7.4) for 2 h. Subsequently, OTCs were rinsed three times with 1x PBS before mounting the tissue on glass coverslips using sRIMS (see also ref. ^71^). For colocalization experiments the same procedure was followed, except CaMello-5HT_2A_ or the 5-HT_2A_ receptor mCherry construct were coexpressed with the corresponding markers and treated as described above under cell culture experiments. For antibody stainings against the endogenous 5-HT_2A_ receptor OTCs were transfected with mCherry at DIV 7. Overexpression was allowed for 24 h. Subsequently, OTCs were fixed with 37 °C PFA (4%) for 30 min, washed, treated with 0.5% Triton and BSA. The primary rabbit anti-5-HT_2A_ receptor antibody (1:1000, ab66049, Abcam) was incubated overnight. On the following day, after multiple washing steps with 1x TBS, the secondary goat anti-rabbit antibody Cy5 (1:500, A10523, Thermo Fisher) was incubated for 1 h. Afterward, OTCs were washed 3x with TBS and 3x with PB before being mounted in sRIMS as described above.

### Statistics

Statistical significance, test procedure and numbers of cells, animals and/or trials performed (*n*) are specified in the figure legends. Statistical significance in all experiments was evaluated using SigmaPlot software (Systat Software) or Igor Pro software (WaveMetrics). For all results, the level of significance was set to *p* < 0.05. Statistical significance is indicated with ***p < 0.001; **p < 0.01; *p < 0.05; n.s. (not significant).

### Reporting summary

Further information on experimental design is available in the Nature Research Reporting Summary linked to this article.

## Supplementary information

Supplementary Information

Reporting Summary

Description of Additional Supplementary Files

Supplementary Video 1

Supplementary Video 2

Supplementary Video 3

Supplementary Video 4

Supplementary Video 5

Supplementary Video 6

Supplementary Data 1

## Data Availability

The authors declare that all the data supporting the findings of this study are available in the manuscript, figures and supplementary information files. All materials and other data supporting this study are readily available from the authors upon reasonable request.
