# Supplementary material for: CaMello-XR enables visualization and optogenetic control of Gq/11 signals and receptor trafficking in GPCR-specific domains
Source: Commun Biol. 2019 Feb 14;2:60. doi: 10.1038/s42003-019-0292-y (PMC6376006; doi:10.1038/s42003-019-0292-y)
Supplement: Supplementary file 1 — Supplementary Information [file 42003_2019_292_MOESM1_ESM.pdf]

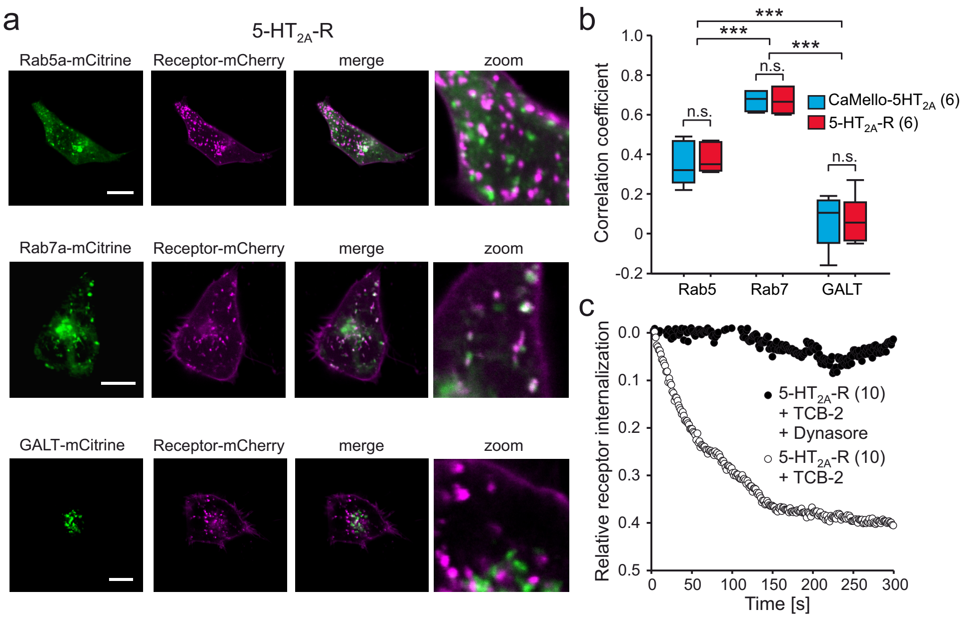

Supplementary Figure 1 Subcellular localization and trafficking of the 5-HT_{2A} receptor in HEK cells. **a** Colocalization of the 5-HT_{2A}-R mCherry construct with early endosomal marker Rab5a (-mCitrine), early to late endosomal marker Rab7a or trans-Golgi network marker GALT (beta-1,4-galactosyltransferase 1) 5 minutes post stimulation with 5-HT_{2A}-R agonist TCB-2 (5 min, 20 μM) in HEK tsA201 cells. Scale bar, 10 μm. **b** Averages of the calculated Pearson's correlation coefficient for CaMello-5HT_{2A} (Fig.1) and the 5-HT_{2A}-R mCherry construct (box plot; one-way analysis of variance (ANOVA) and Holm-Sidak multiple comparison method; n = 6 individual cells for each colocalization pairing; n.s. = not significant, ***$p < 0.001$; p left to right within groups: 0.930, 1.000, 0.995; p between groups: < 0.001). **c** Time course of activation-dependent receptor internalization for the 5-HT_{2A}-R mCherry construct. Mean (n = 10 dishes) traces of normalized activation-dependent receptor internalization monitored via differences in reduction of membrane-localized mCherry fluorescence between stimulated (561 nm + TCB-2 20 μM) and unstimulated (561 nm) trials with and without addition of dynamin inhibitor Dynasore (50 μM).

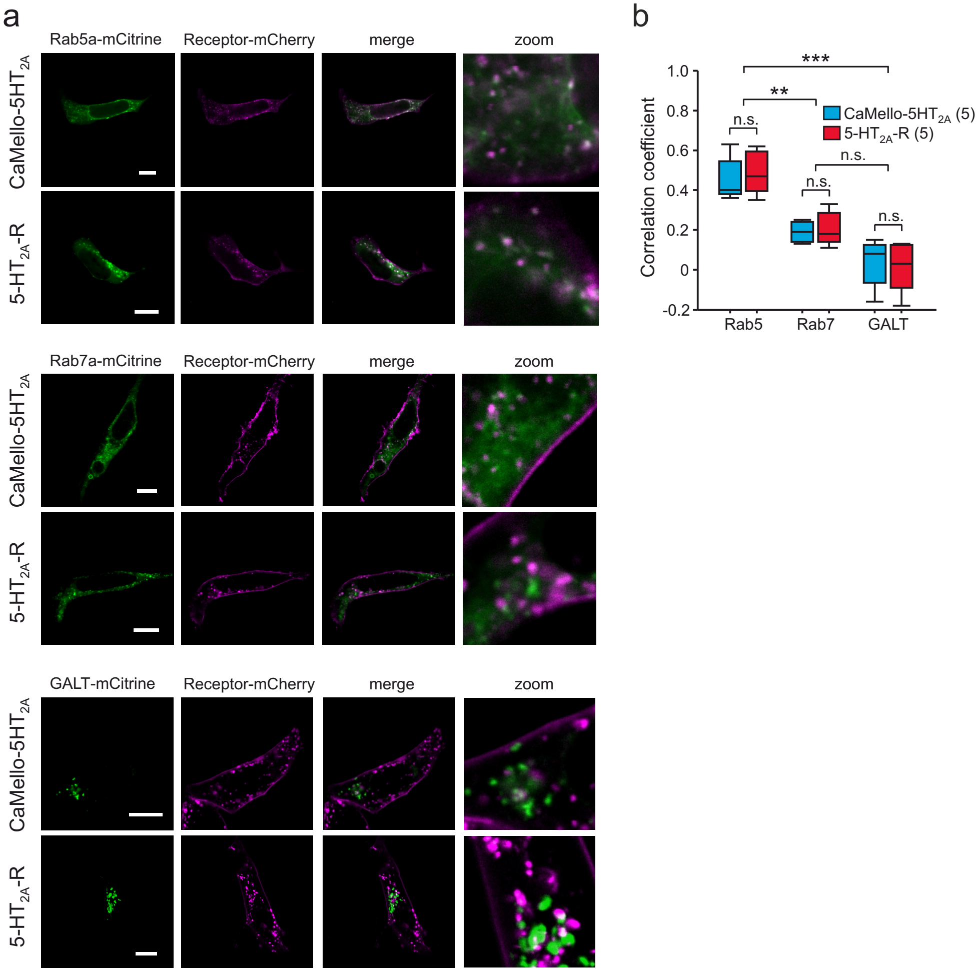

Supplementary Figure 2 Subcellular localization and trafficking of CaMello-5HT$_{2A}$ and the 5-HT$_{2A}$ receptor in HEK cells following low intensity light or 5-HT stimulation. **a** Colocalization of CaMello-5HT$_{2A}$ and the 5-HT$_{2A}$-R mCherry construct with early endosomal marker Rab5a (-mCitrine), early to late endosomal marker Rab7a or trans-Golgi network marker GALT (beta-1,4-galactosyltransferase 1) 5 minutes post stimulation with low intensity 476 nm light (5min) or 1 μM 5-HT (5min) in HEK tsA201 cells. Scale bar, 10 μm. **b** Averages of the calculated Pearson's correlation coefficient for CaMello-5HT$_{2A}$ and the 5-HT$_{2A}$-R mCherry construct (box plot; one-way analysis of variance (ANOVA) and Holm-Sidak multiple comparison method; n = 5 individual cells for each colocalization pairing; n.s. = not significant, $^{**}p < 0.01$, $^{***}p < 0.001$; p left to right within groups: 0.903, 0.806, 0.942; p left to right between groups: 0.004, 0.002, 0.110, 0.056, < 0.001, < 0.001).

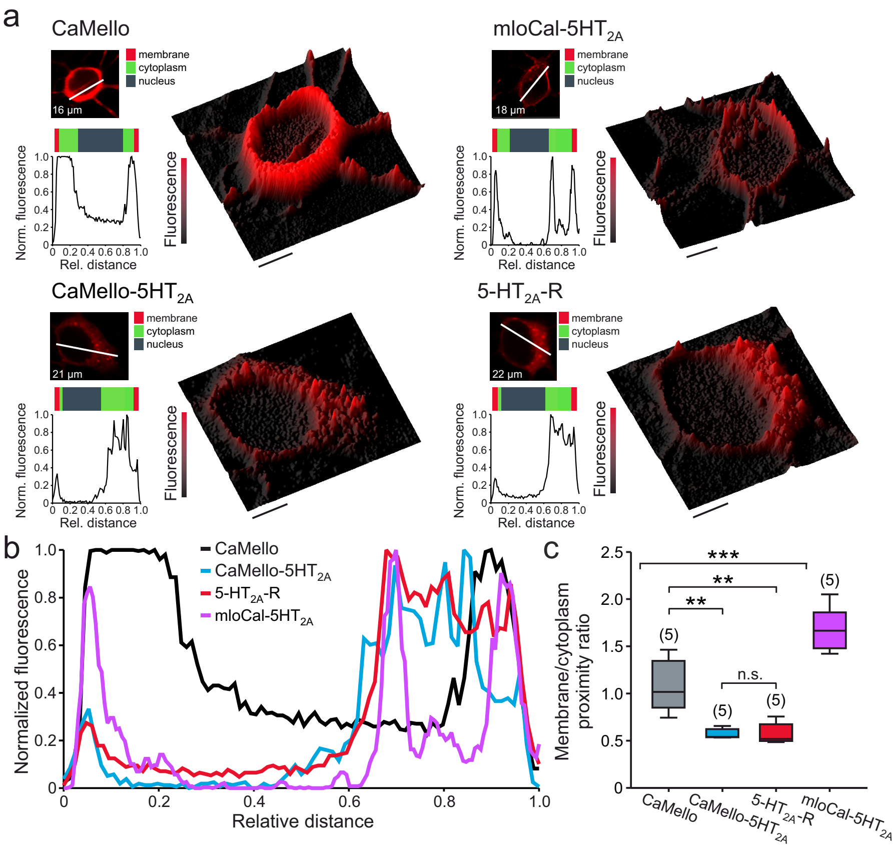

Supplementary Figure 3 Subcellular distribution and membrane proximity of CaMello, CaMello-5HT$_{2A}$, 5-HT$_{2A}$-R-mCherry and mloCal-5HT$_{2A}$ in rat visual cortex organotypic cultures (OTCs). **a** Close-up confocal z-section images of transfected cell somata were visualized using the mCherry reporter (images). Quantitative analysis of the fluorescence intensity for each individual construct along the white line in the image panels with indication of membrane, cytoplasm and nucleus areas of the corresponding neuron, evaluated via z-section reconstruction (graph). 3D surface plots depicting the quantitative fluorescence intensity throughout the depicted z-sections of the analyzed neuronal somata (plots). Scale bar, 5 µm. **b** Merge of the quantitative fluorescence intensity analysis for CaMello, CaMello-5HT$_{2A}$, 5-HT$_{2A}$-R-mCherry and mloCal-5HT$_{2A}$ as shown in a. **c** Comparison of relative membrane to cytoplasm proximity ratio for CaMello, CaMello-5HT$_{2A}$, 5-HT$_{2A}$-R-mCherry and mloCal-5HT$_{2A}$ via quantitative calculation of average membrane and cytoplasm fluorescence intensity depending on proximity to the cellular membrane, evaluated via z-section reconstruction ((box plot; one-way analysis of variance (ANOVA) and Holm-Sidak multiple comparison method; n = 5 individual cells, pooled from 2 animals per group; n.s. = not significant, **p < 0.01, ***p < 0.001: p left to right: 0.002, 0.001, 0.998, < 0.001, < 0.001, < 0.001).

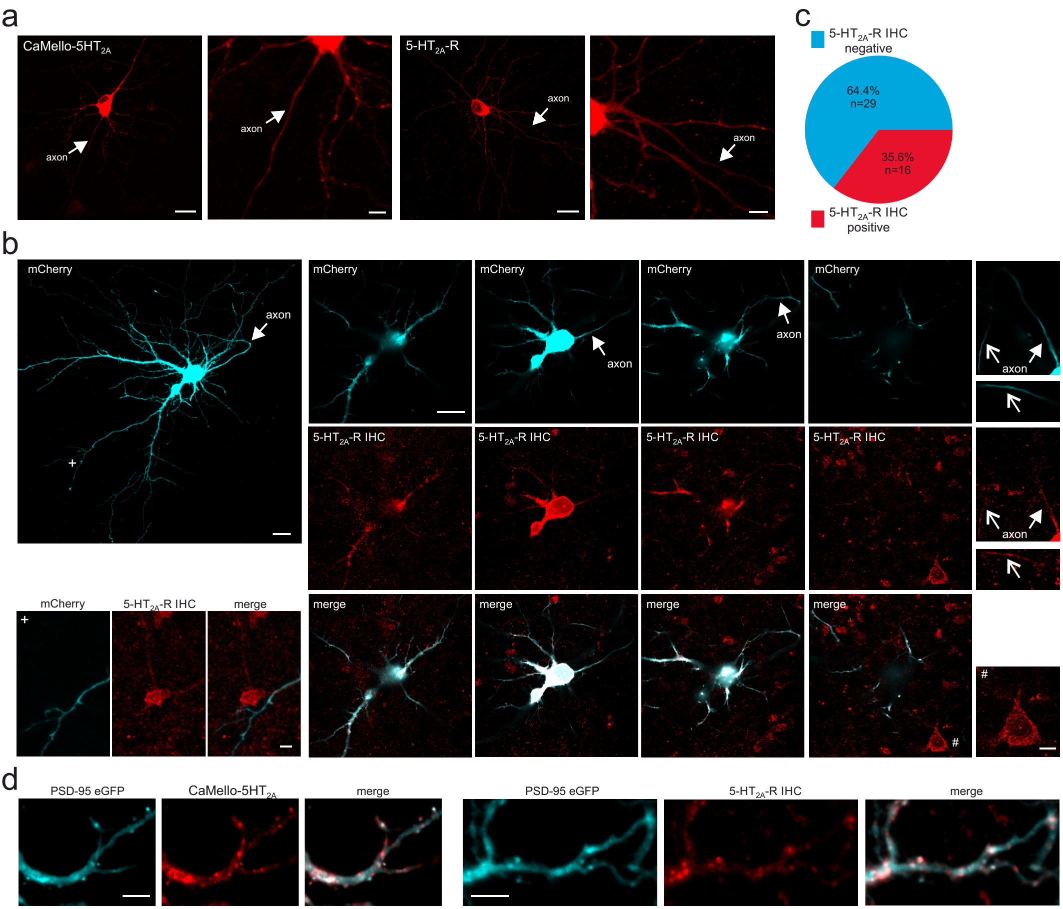

Supplementary Figure 4 Subcellular localization and trafficking of the 5-HT_{2A} receptor in rat visual cortex organotypic cultures (OTCs). **a** Expression pattern of CaMello-5HT_{2A} and the 5-HT_{2A} receptor mCherry construct (5HT_{2A}-R) in rat cortical neurons (OTCs) (white arrows indicate the axon). Scale bar, 20 µm overview, 5 µm zoom. **b** Antibody staining against the endogenous 5-HT_{2A} receptor (5-HT_{2A}-R IHC, IHC = immunohistochemistry) (red) in OTC neurons randomly expressing mCherry (green) via gene gun transfection (white arrows indicate the axon) (images). Note, somatic expression is overexposed to visualize dendritic and axonal expression patterns. Scale bar, 20 µm overview, 5 µm + and #. **c** Relative proportion of 5-HT_{2A} receptor IHC positive neurons (n = 45 individual cells pooled from 5 animals) (pie chart). **d** Coexpression of CaMello-5HT_{2A} and postsynaptic density protein 95 (PSD-95 eGFP) in OTC neurons (left) and PSD-95 eGFP together with 5-HT_{2A}-R IHC (right). Scale bar, 5 µm.

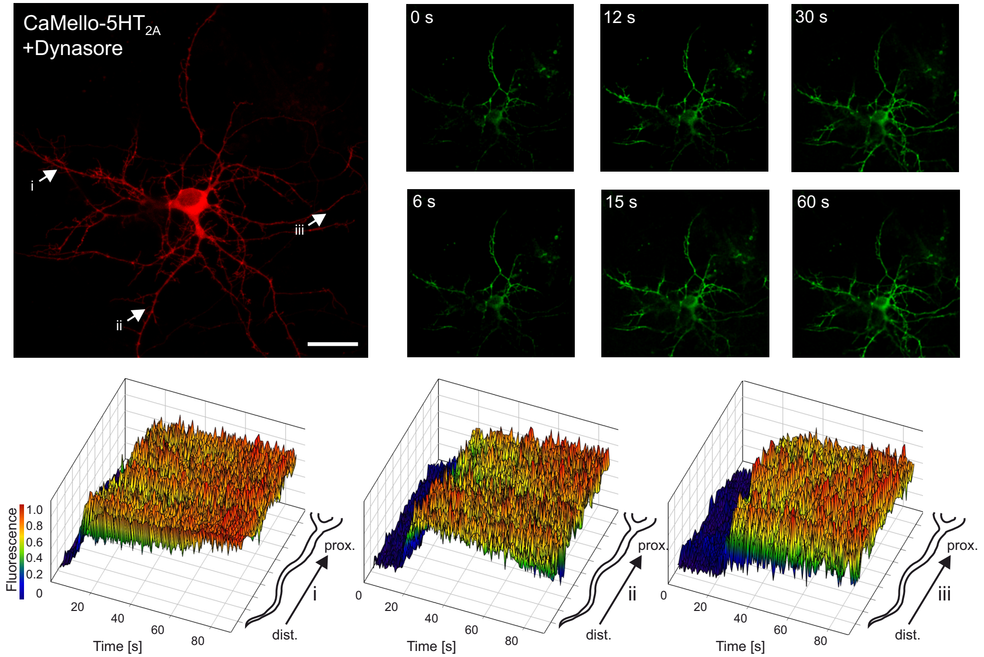

Supplementary Figure 5 Time course of light-induced Ca^{2+} responses in rat visual cortex OTCs for CaMello-$5HT_{2A}$ under internalization block via dynamin inhibitor Dynasore (50 µM). Transfected cells were visualized using the mCherry reporter and Ca^{2+} signals were light-induced (476 nm + 495 nm) and measured via GCaMP6m monitoring (images). 3D mesh plots of individual neurites (i, ii, iii) showing normalized light-induced Ca^{2+} responses during 90 s of illumination from distal to proximal (plots). Scale bar, 20 µm.

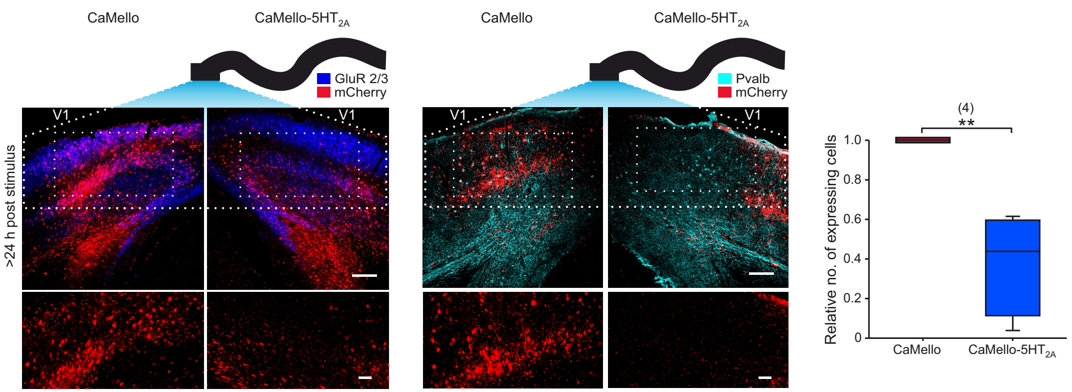

Supplementary Figure 6 Coronal images depicting brain sections of the visual cortex expressing CaMello and CaMello-5HT$_{2A}$ for animals > 24 h after photostimulation (related to Figure 6). Pyramidal neurons were antibody-stained against GluR2/3, while Pvalb+ neurons were stained against parvalbumin. (GluR: glutamate receptor; Pvalb: parvalbumin) (images). The relative number of expressing cells in the illuminated area was analyzed and animals were killed >24 h after stimulation (box plot; unpaired t-test; n = 4 animals per group; $^{**}p < 0.01$; $p = 0.002$) (box plot). Scale bar, 150 µm overview, 50 µm zoom.
