# Supplementary material for: CaMello-XR enables visualization and optogenetic control of Gq/11 signals and receptor trafficking in GPCR-specific domains
Source: Commun Biol. 2019 Feb 14;2:60. doi: 10.1038/s42003-019-0292-y (PMC6376006; doi:10.1038/s42003-019-0292-y)
Supplement: Supplementary file 2 — Reporting Summary [file 42003_2019_292_MOESM2_ESM.pdf]

nature research

Corresponding author(s): Stefan Herlitze

Last updated by author(s): Dec 20, 2018

Reporting Summary

Nature Research wishes to improve the reproducibility of the work that we publish. This form provides structure for consistency and transparency in reporting. For further information on Nature Research policies, see Authors & Referees and the Editorial Policy Checklist.

Statistics

For all statistical analyses, confirm that the following items are present in the figure legend, table legend, main text, or Methods section.

n/a	Confirmed	
☐	☒	The exact sample size (*n*) for each experimental group/condition, given as a discrete number and unit of measurement
☐	☒	A statement on whether measurements were taken from distinct samples or whether the same sample was measured repeatedly
☐	☒	The statistical test(s) used AND whether they are one- or two-sided<br>*Only common tests should be described solely by name; describe more complex techniques in the Methods section.*
☒	☐	A description of all covariates tested
☐	☒	A description of any assumptions or corrections, such as tests of normality and adjustment for multiple comparisons
☐	☒	A full description of the statistical parameters including central tendency (e.g. means) or other basic estimates (e.g. regression coefficient) AND variation (e.g. standard deviation) or associated estimates of uncertainty (e.g. confidence intervals)
☐	☒	For null hypothesis testing, the test statistic (e.g. *F*, *t*, *r*) with confidence intervals, effect sizes, degrees of freedom and *P* value noted<br>*Give P values as exact values whenever suitable.*
☒	☐	For Bayesian analysis, information on the choice of priors and Markov chain Monte Carlo settings
☒	☐	For hierarchical and complex designs, identification of the appropriate level for tests and full reporting of outcomes
☒	☐	Estimates of effect sizes (e.g. Cohen's *d*, Pearson's *r*), indicating how they were calculated

Our web collection on statistics for biologists contains articles on many of the points above.

Software and code

Policy information about availability of computer code

Data collection	ImageJ (Fiji contributors version); Leica Application Suite (for Leica TCS SP5 confocal laser scanning microscope); HEKA PatchMaster software (v2x90.3);
Data analysis	ImageJ (Fiji contributors version); Wavemetrics Igor Pro Software (v6.0); Originlab Origin Pro (v8); Microsoft Excel (2016); Systat Software Sigmaplot (v12.5);

For manuscripts utilizing custom algorithms or software that are central to the research but not yet described in published literature, software must be made available to editors/reviewers. We strongly encourage code deposition in a community repository (e.g. GitHub). See the Nature Research guidelines for submitting code & software for further information.

Data

Policy information about availability of data

All manuscripts must include a data availability statement. This statement should provide the following information, where applicable:

- Accession codes, unique identifiers, or web links for publicly available datasets
- A list of figures that have associated raw data
- A description of any restrictions on data availability

The authors declare that all the data supporting the findings of this study are available in the manuscript, figures and supplementary information files. All materials and other data supporting this study are readily available from the authors upon reasonable request.

Field-specific reporting

Please select the one below that is the best fit for your research. If you are not sure, read the appropriate sections before making your selection.

☒ Life sciences ☐ Behavioural & social sciences ☐ Ecological, evolutionary & environmental sciences

For a reference copy of the document with all sections, see nature.com/documents/nr-reporting-summary-flat.pdf

Life sciences study design

All studies must disclose on these points even when the disclosure is negative.

Sample size	Sample size was estimated with GPower 3.1
Data exclusions	No data were excluded from the analyses
Replication	All attempts at replication were successfull
Randomization	Allocation of samples into experimental groups was random
Blinding	Investigators were blinded during experiments (e.g. they did not know which construct was currently expressed/tested)

Reporting for specific materials, systems and methods

We require information from authors about some types of materials, experimental systems and methods used in many studies. Here, indicate whether each material, system or method listed is relevant to your study. If you are not sure if a list item applies to your research, read the appropriate section before selecting a response.

Materials & experimental systems

n/a	Involved in the study	
☐	☒	Antibodies
☐	☒	Eukaryotic cell lines
☒	☐	Palaeontology
☐	☒	Animals and other organisms
☒	☐	Human research participants
☒	☐	Clinical data

Methods

n/a	Involved in the study	
☒	☐	ChIP-seq
☒	☐	Flow cytometry
☒	☐	MRI-based neuroimaging

Antibodies

Antibodies used	rabbit anti-GluR 2/3 (primary, 1:300, 07-598, Merck); mouse anti-PV (primary, 1:1000, P3088, Sigma-Aldrich); mouse anti-Calbindin (primary, 1:500, MO19000, Neuromics); goat anti-rabbit Cy5 (secondary, 1:500, 711-175-152, Jackson ImmunoResearch); donkey anti-mouse DyLight 650 (secondary, 1:500, SA5-10169, Thermo Fisher); goat anti-SR-2A (primary, 1:200, SC-15074, Santa Cruz); donkey anti-goat Alexa 633 (secondary, 1:500, A-21082, Thermo Fisher);
Validation	rabbit anti-GluR 2/3 (primary, 1:300, 07-598, Merck): http://www.merckmillipore.com/DE/de/product/Anti-GluR2/3-Antibody,MM_NF-07-598#overview<br><br>mouse anti-PV (primary, 1:1000, P3088, Sigma-Aldrich): https://www.sigmaaldrich.com/catalog/product/sigma/p3088?<br><br>mouse anti-Calbindin (primary, 1:500, MO19000 Neuromics): http://biohj.com/proshow.aspx?id=152963<br><br>goat anti-SR-2A (primary, 1:200, SC-15074, Santa Cruz): https://www.scbt.com/scbt/product/sr-2a-antibody-h-18?

Eukaryotic cell lines

Policy information about cell lines

Cell line source(s)	HEK tsa201 Cell Line Human (SIGMA-ALDRICH); HEK GIRK 1/2 cells (kindly provided by Dr. A. Tinker UCL London, GB)
Authentication	Cell line authentication was conducted by supplier (SIGMA-ALDRICH)
Mycoplasma contamination	All cell lines tested negative for mycoplasma contamination

Commonly misidentified lines (See ICLAC register)	*Name any commonly misidentified cell lines used in the study and provide a rationale for their use.*

Animals and other organisms

Policy information about studies involving animals; ARRIVE guidelines recommended for reporting animal research

Laboratory animals	Long Evans rats, neonatal, male (for OTC culture generation); C57Bl/6J mice, adult (1-3 months), male
Wild animals	The study did not involve wild animals
Field-collected samples	The study did not involve field-collected samples
Ethics oversight	All animal experiments were approved by the Institutional Animal Research Facility (Ruhr-University Bochum; specialist contact person: animal welfare officer PD Dr. Matthias Schmidt)

Note that full information on the approval of the study protocol must also be provided in the manuscript.
