# Supplementary material for: CaMello-XR enables visualization and optogenetic control of Gq/11 signals and receptor trafficking in GPCR-specific domains
Source: Commun Biol. 2019 Feb 14;2:60. doi: 10.1038/s42003-019-0292-y (PMC6376006; doi:10.1038/s42003-019-0292-y)
Supplement: Supplementary file 3 — Description of Additional Supplementary Files [file 42003_2019_292_MOESM3_ESM.docx]

**Supplementary Data 1:** Source data underlying the main article figures.

**Supplementary Video 1/2** (related to Figure 1) Optogenetic control and visualization of calcium signals with CaMello-XRs. Compressed videos depicting the time course of light-induced Ca^2+^ responses for CaMello (S1) and CaMello-5HT_2A_ (S2) in HEK tsA 201 cells for the cells shown in Figure 1. Light-induced calcium signals were measured via GCaMP6m monitoring for 60 s (476 + 495 nm).

**Supplementary Video 3/4/5/6** (related to Figure 3) Optogenetic control and visualization of local calcium signals in rat visual cortex organotypic cultures (OTCs). Compressed videos depicting the time course of light-induced (CaMello (S3) + CaMello-5HT_2A_ (S4)) and agonist-induced (5-HT_2A_ receptor (S5)) local Ca^2+^ responses in rat visual cortex OTCs for the cells shown in Figure 3. Light- and agonist-induced calcium signals were measured via GCaMP6m monitoring for 90 s (476 + 495 nm). Animated 3D surface plots (S6) depicting the time course of light-induced (CaMello + CaMello-5HT_2A_) and agonist-induced (5-HT_2A_ receptor) local Ca^2+^ responses in selected neurite regions of rat visual cortex OTCs as seen in Figure 3 and Videos S4/5/6. Light- and agonist-induced calcium signals were measured via GCaMP6m monitoring for 90 s (476 + 495 nm) and fluorescence intensity was plotted over time.
